# Flow Cytometry-Based Measurement of Antibodies Specific for Cell Surface-Expressed Folded SARS-CoV-2 Receptor-Binding Domains

**DOI:** 10.3390/vaccines12040377

**Published:** 2024-04-01

**Authors:** Al Nasar Ahmed Sehgal, Jera Safran, Bernhard Kratzer, Pia Gattinger, Robert B. Stieger, Laszlo Musiejovsky, Doris Trapin, Paul Ettel, Ulrike Körmöczi, Arno Rottal, Kristina Borochova, Yulia Dorofeeva, Inna Tulaeva, Milena Weber, Katharina Grabmeier-Pfistershammer, Thomas Perkmann, Ursula Wiedermann, Rudolf Valenta, Winfried F. Pickl

**Affiliations:** 1 Institute of Immunology, Center for Pathophysiology, Infectiology and Immunology, Medical University of Vienna, 1090 Vienna, Austrian11917171@students.meduniwien.ac.at (J.S.); 11774079@mail.sfu.ac.at (R.B.S.);; 2 Institute of Pathophysiology and Allergy Research, Center for Pathophysiology, Infectiology and Immunology, Medical University of Vienna, 1090 Vienna, Austria; 3 Laboratory for Immunopathology, Department of Clinical Immunology and Allergology, Sechenov First Moscow State Medical University, 119991 Moscow, Russia; 4 Department of Laboratory Medicine, Medical University of Vienna, 1090 Vienna, Austria; thomas.perkmann@meduniwien.ac.at; 5 Institute of Specific Prophylaxis and Tropical Medicine, Center for Pathophysiology, Infectiology and Immunology, Medical University of Vienna, 1090 Vienna, Austria; 6 NRC Institute of Immunology FMBA of Russia, 115478 Moscow, Russia; 7 Karl Landsteiner University of Health Sciences, 3500 Krems, Austria

**Keywords:** SARS-CoV-2, COVID-19, antibody response, SARS-CoV-2 immunity, flow-cytometry, serodiagnosis, HEK-293T cell lines, RBD

## Abstract

Background: COVID-19, caused by severe acute respiratory syndrome coronavirus 2 (SARS-CoV-2), has now become endemic and is currently one of the important respiratory virus infections regularly affecting mankind. The assessment of immunity against SARS-CoV-2 and its variants is important for guiding active and passive immunization and SARS-CoV-2-specific treatment strategies. Methods: We here devised a novel flow cytometry-based diagnostic platform for the assessment of immunity against cell-bound virus antigens. This platform is based on a collection of HEK-293T cell lines which, as exemplified in our study, stably express the receptor-binding domains (RBDs) of the SARS-CoV-2 S-proteins of eight major SARS-CoV-2 variants, ranging from Wuhan-Hu-1 to Omicron. Results: RBD-expressing cell lines stably display comparable levels of RBD on the surface of HEK-293T cells, as shown with anti-FLAG-tag antibodies directed against a N-terminally introduced 3x-FLAG sequence while the functionality of RBD was proven by ACE2 binding. We exemplify the usefulness and specificity of the cell-based test by direct binding of IgG and IgA antibodies of SARS-CoV-2-exposed and/or vaccinated individuals in which the assay shows a wide linear performance range both at very low and very high serum antibody concentrations. In another application, i.e., antibody adsorption studies, the test proved to be a powerful tool for measuring the ratios of individual variant-specific antibodies. Conclusion: We have established a toolbox for measuring SARS-CoV-2-specific immunity against cell-bound virus antigens, which may be considered as an important addition to the armamentarium of SARS-CoV-2-specific diagnostic tests, allowing flexible and quick adaptation to new variants of concern.

## 1. Introduction

After the initial appearance of the severe acute respiratory syndrome coronavirus 2 (SARS-CoV-2) in December 2019, the COVID-19 pandemic has spread worldwide. In the meantime, COVID-19 has become a part of the respiratory viral infectious diseases continuously affecting the world population, with high peaks during the common cold seasons. Despite the presence of a proof-reading exonuclease protein, which is atypical of many other RNA viruses [1], this single-stranded, positive-sense RNA virus has accumulated remarkable numbers of mutations in a very short time, leading to antigenic drift and the emergence of new variants of concern (VOCs). The World Health Organization (WHO) has designated several VOCs, with Alpha, Beta, Gamma, Delta, and Omicron (https://www.who.int/publications/m/item/historical-working-definitions-and-primary-actions-for-sars-cov-2-variants (accessed on 31 November 2023)) and four potential sub-lineages of Omicron VOC currently ranked as variants of interest (VOIs) (https://www.who.int/activities/tracking-SARS-CoV-2-variants (accessed on 13 December 2023)). The emergence of new VOCs, which may escape surveillance and defense by SARS-CoV-2-specific immunity, is a topic of high importance. Accordingly, the detection of such variants and the measurement of SARS-CoV-2-cross-protective immunity in the general population and especially in vulnerable groups is a priority. There are currently multiple tests available that allow to measure SARS-CoV-2-specific humoral and cellular immune responses. For example, serological assays, including enzyme-linked immunosorbent assays (ELISA), chemiluminescent assays (ChLIA), meso scale discovery (MSD), rapid serological tests (RST), virus neutralization, and hemagglutination-based tests (HAT), can be performed with sera from COVID-19 convalescent and vaccinated individuals ([2] and reviewed in [3,4,5]). Numerous research studies have substantiated varying degrees of antibody reactivity toward viral variants, with covariates being sample timing, age, and immune status in general, and the severity of disease in recovered individuals as well as the number of doses, type, and regimen of vaccine(s) administered in the vaccinated population in particular [6,7,8,9,10,11]. Of major interest are diagnostic tests that allow to determine the neutralizing potential of SARS-CoV-2-specific antibodies, because such antibodies can protect the host from becoming infected [12,13,14]. Different forms of virus-neutralization tests and molecular interaction assays have been developed that can be used to measure the ability of antibodies to prevent the binding of the receptor-binding domain of the virus, RBD, with its cognate receptor on human cells, ACE2 [3,15]. These assays are a bit more cumbersome than tests measuring the mere presence of SARS-CoV-2-specific antibodies. However, it turns out that mainly antibodies directed to correctly folded RBD are important for virus neutralization [16]. Accordingly, antibodies directed to folded RBD are a useful marker for neutralizing antibodies. While neutralizing IgG antibodies may provide long-lasting protection, the presence of substantial amounts of protective IgA antibodies in the mucosa may be helpful for the neutralization of respiratory viruses such as SARS-CoV-2.

We hypothesized that test platforms based on cells stably expressing virus antigens can be useful to measure antibody responses, both IgG and IgA, against folded RBD and, if this can be demonstrated, eventually also for measuring SARS-CoV-2-specific cellular immunity e.g., antibody-dependent cellular cytotoxicity (ADCC).

In this study, we have made a first step toward this direction and established stably transduced HEK-293T cell lines expressing the RBDs of the spike (S) proteins of several SARS-CoV-2 variants on the cell surface. The herein-established flow cytometry-based cellular assay (FCCA) has been evaluated with the sera of ten different cohorts of individuals, varying by parameters such as COVID-19 convalescence and different vaccination regimens. Moreover, a SARS-CoV-2 non-exposed control group was evaluated in parallel. The FCCA has shown to be very versatile because it could be easily equipped with RBDs from different virus variants. It demonstrated improved resolution, a wide linear performance range, a strong positive correlation with RBD-based ELISA, and remarkable sensitivity and specificity. Moreover, RBD variant-expressing HEK-293T cells proved to be highly useful as an adsorption matrix to determine the variant specificity of RBD-binding antibodies in sera.

## 2. Materials and Methods

Unless indicated otherwise, chemicals were purchased from Sigma-Aldrich, St. Louis, MO, USA. For simplicity in figures, we refer to Wuhan-Hu-1 as Wuhan and Omicron BA.1 as Omicron.

### 2.1. Patients and Control Subjects

Venous blood was drawn from all subjects and either plasma from heparin-anticoagulation tubes or serum from silicon dioxide coagulation tubes was isolated and the aliquots were kept at −80 °C until further use. Details on the recruitment of patients can be found in [12,17]. Briefly, the non-exposed individuals (group A), sampled between May and June 2020, were reportedly asymptomatic for the last 10 weeks and were SARS-CoV-2 negative as revealed by a certified SARS-CoV-2 NC-based antibody test (Elecsys^®^ Anti-SARS-CoV-2 assay Roche) and rtPCR and/or ELISA-based antigen test at the time of venipuncture. Convalescent patients (groups B1–B4 and E) had rtPCR-confirmed COVID-19 disease, while the vaccination status (groups B3–E) was individually reported by each patient. Time to the ‘last event’, defined as infection or vaccination, was set ≥ 20 days as the uniform inclusion criterion across all groups except for the healthy controls. Table 1 shows the grouping of individuals along with their basic demographic data into 11 groups according to their infection and vaccination status. All patients gave their written informed consent to participate in the study in accordance with the Declaration of Helsinki. The study was approved by the Ethics Committee of the Medical University of Vienna (EK No.: 1302/2020).

### 2.2. Generation of SARS-CoV-2 RBD (and Spike) Expression Constructs

The protein sequence of RBD of the S protein of SARS-CoV-2 was taken from the Wuhan-Hu-1 isolate, Genbank accession no. QHD43416.1, amino acids 330–583 [12,18,19]. The bi-cistronic expression construct, PPT::FLAG::RBD::CD16b-GPI::IRES::Puro (Table 2), which was codon-optimized for expression in HEK-293T cells, started with the pre-pro-trypsin signal peptide sequence [20] followed by a 3×-tandem FLAG tag ([21,22] and https://www.sigmaaldrich.com/AT/de/technical-documents/technical-article/genomics/cloning-and-expression/3x-flag (accessed on 29 October 2021)), a G_4_S linker in between, and RBD, which was fused at its C-terminus to the minimal human CD16b-GPI anchor acceptor sequence, taken from GenBank no. X07934.1, amino acids 193–233 [23]. It was introduced into the lentiviral pHR transfer vector [24] by directional cloning using XhoI and NotI restriction enzymes. Downstream, a puromycin resistance gene translated in a cap-independent manner through the internal ribosome entry site (IRES) (ATG:biosynthetics GmbH, Merzhausen, Germany) was cloned with NotI and MluI (Figure S1) [25]. For easier handling, variant-specific point mutations were introduced into RBDs with the constructs being located in the pEAK12 expression vector (Edge Biosystems, Gaithersburg, MD, USA), followed by swapping of the sequence-verified, mutated expression cassettes into pHR. The indicated point mutations (Figure S2) were introduced using the Q5^®^ Site-Directed Mutagenesis Kit (New England BioLabs GmBH, Frankfurt, Germany) along with the primer pairs listed in Table S1, except for the RBD-Omicron DNA sequence, which was synthesized and subcloned into pHR (ATG:biosynthetics GmbH) (Table S2). The accuracy of all resulting transfer vectors, pHR_PPT::FLAG::RBD::CD16b-GPI::IRES::Puro, was validated by restriction analysis using XhoI-NotI and NotI-MluI endonucleases, respectively, and by double-strand sequencing (Eurofins Genomics GmbH, Cologne, Germany). The SARS-CoV-2 Spike-Wuhan-Hu-1 expression construct containing the complete spike protein sequence, Genbank accession no. QHR63270.2, amino acids 13–1213, (60-aa truncated from the C-terminus, representing part of the transmembrane domain and cytoplasmic tail), was commercially synthesized in the pEAK12 cloning vector as PPT::FLAG::Spike::CD16b-GPI (ATG:biosynthetics) (Table 2), subcloned into the lentiviral pHR vector using XhoI and NotI, resulting in pHR_PPT::FLAG::S::CD16b-GPI, and then sequence verified, as described above. Restriction endonucleases were purchased from New England BioLabs.

### 2.3. Development of Stable Cell Lines

Amphotropic lentiviruses were produced by transient transfection using an optimized calcium-phosphate coprecipitation method [27]. Briefly, 1 × 10^6^ HEK-293T cells were seeded in a 10 cm tissue culture dish (Sarstedt, Nümbrecht, Germany) one day prior to transfection with a replication-incompetent 2^nd^ generation lentivirus system. Aliquots of 15 µg pHR_PPT::FLAG::RBD::CD16b-GPI::IRES::Puro or 15 µg pHR_GFP, used as transfection control, were mixed with 10 µg pS_Pax2 [24] (kindly provided by Dr. P. Steinberger, Medical University of Vienna, Vienna, Austria) and 5 µg pMD_VSV-G [28] (kindly provided by Dr. R. C. Mulligan, Harvard Medical School, Boston, MA, USA). The medium containing viral particles was harvested and filtered (0.45 µm) after 72 hours post-transfection and subjected to 10× concentration with PEG-it™ Virus Precipitation Solution (System Biosciences, Mountain View, CA, USA) as per the manufacturer’s protocol. Aliquots of 3 × 10^5^ HEK-293T cells were seeded in a flat-bottom standard 6-well plate (Sarstedt) in 1.8 mL of medium one day before they were transduced with 200 µL of 10-fold concentrated viral particles in the presence of 8 µg/mL polybrene and spinoculated with 670× *g* at 32 °C for 90 min. At least 12 single-cell clones (SCCs) for each RBD variant construct were established after low-density seeding of transduced cells in the medium containing 4 µg/mL puromycin. The clones with the highest RBD expression level, based on anti-FLAG-tag immunofluorescence staining (Table S3), were selected for further studies. For the generation of SARS-CoV-2 Spike (Wuhan-Hu-1)-expressing HEK-293T cell line, the pHR_PPT::FLAG::Spike::CD16-GPI vector was used and transfection, transduction, and single-cell cloning were performed, as described above. HEK-293T pHR_LgBiT puromycin-resistant cells (Promega Corporation, Madison, WI, USA) were used as a control for pHR-transduced HEK-293T cells. Parental HEK-293T(-p) and Spike-HEK-293T cell lines were passaged in Hyclone IMDM-modified medium (Cytiva, Pasching, Austria) supplemented with 10% heat-inactivated FBS (Gibco, Life Technologies Corporation, Grand Island, NY, USA) and 15 µg/mL gentamicin, while 4 µg/mL puromycin was included for the culture of pHR_PPT::FLAG::RBD::CD16-GPI::IRES::Puro transduced cells. Prior to anti-FLAG staining, cell lines were grown in the medium supplemented with 50 mM NaClO_3_ for 48 h [29,30]. From all cell lines established as single-cell clones (SCCs), aliquots of master stocks were cryopreserved.

### 2.4. Flow Cytometry-Based Cellular Assay (FCCA)

HEK-293T SCCs expressing the SARS-CoV-2 RBD variants were harvested 72 h after seeding from standard 150 mm tissue culture dishes (Sarstedt) with 10 mL of cold PBS (1×) (without Ca^2+^, Mg^2+^) (Gibco, Life Technologies Corporation). The cell pellet, obtained by centrifugation at 500× *g* for 5 min, was resuspended in 5 mL of cold PBS (1×) (without Ca^2+^, Mg^2+^) and the cell number was determined with a Coulter Counter Z2 (Beckmann Coulter, Brea, CA, USA). Cells were then washed twice with cold PBS (1×) and adjusted to a concentration of 4 × 10^6^ cells/mL. For determination of cell viability, 2 µL of Aqua Zombie (BioLegend Europe B.V, Amsterdam, The Netherlands) was added to 4 × 10^6^ cell suspension, which subsequently was incubated at room temperature for 10 min. The reaction was stopped by washing the cells with 25 mL of FACS buffer (1× PBS, 0.5% BSA, 2 mM EDTA, and 0.05% NaN_3_). For immunofluorescence staining, aliquots of 2 × 10^5^ cells in 50 µL FACS buffer were added to 4.5 mL polystyrene tubes (Becton Dickinson, Franklin lakes, NJ, USA), followed by 20 µL of 1:100 diluted serum/plasma sample in FACS buffer, and incubated at 4 °C for 30 min. After washing with 4 mL of FACS buffer, 20 µL of a mixture of APC-conjugated anti-human IgG and PE-conjugated anti-human IgA secondary antibodies was added at dilutions listed in Table S4 and incubated at 4 °C for 30 min. After this final incubation, cells were washed again with 4 mL of FACS buffer and the supernatant was discarded. Samples of 1 × 10^4^ Aqua Zombie-negative singlets were acquired on a CytoFLEX S flow cytometer (Beckman Coulter GmbH, Vienna, Austria) equipped with the CytExpert software package (version 2.4) and analyzed with FlowJo version 10.8.1 (Becton Dickinson). To maintain consistency and accuracy, quality control was performed using CytoFLEX Daily QC beads (Beckman Coulter) and identical gain settings were ensured before the acquisition of each batch. Moreover, single-color compensation controls were run and the calculated compensation matrix was manually verified before applying it to the data obtained by the FCCA. The staining with each serum sample was performed in duplicate and the specific signal (change in geometric mean fluorescence intensity), i.e., ∆gMFI, was calculated by subtracting the background gMFI obtained upon staining of the HEK-293T-p cells in the presence of sample plus fluorescently labeled secondary antibodies. To further account for the interassay variability, the FCCA thresholds for IgG and IgA antibodies were fixed at one standard deviation (SD) of the mean gMFI value obtained with the respective fluorescently labeled secondary antibodies in the absence of serum in 18 independent experiments performed with all nine cell lines (each cell line tested at least three times), which amounted to log_10_(1372) = 3.14 for IgG, and log_10_(851) = 2.93 for IgA. For the assessment of ACE2 binding, the HEK-293T-RBD-Wuhan-Hu-1 cell line and the parental HEK-293T cells were incubated with 1:650 diluted human ACE2-hFc protein (GenScript Biotech, Rijswijk, The Netherlands) and, after a washing step, probed with PE-conjugated anti-human IgG antibody (Jackson ImmunoResearch, Ely, Cambridgeshire, UK) at 1:100 dilution to detect the ACE2-hFc portion. The samples were acquired and analyzed in a similar fashion.

### 2.5. Enzyme-Linked Immunosorbent Assay (ELISA)

A previously established ELISA protocol for the detection of anti-RBD SARS-CoV-2 antibodies was adapted [12]. Briefly, 100 µL of recombinant SARS-CoV-2 RBD-Wuhan-Hu-1-Avi-His tag, 319–591 aa, (GenScript Biotech), RBD-Delta-His tag or RBD-Omicron BA.1-His tag (Sino Biological Europe GmbH, Eschborn, Germany), 319–541 aa, were coated at a concentration of 2 µg/mL in bicarbonate buffer on 96-well flat-bottom high-binding microplates (Corning Life Science, Glendale, AZ, USA) at 4 °C overnight. The next day, the plates were washed six times with 300 µL/well of PBS-T (0.1% Tween-20, Bio-Rad Laboratories, Hercules, CA, USA) and blocked with PBS-T containing 3% BSA at room temperature for 3 h. For the determination of specific IgG levels, 100 µL of 1:200 diluted serum samples in dilution buffer (PBS-T plus 1% BSA) were added per well and incubated at 4 °C overnight. On the following day, the plates were washed six times with a microplate washer (Agilent BioTeK, Santa Clara, CA, USA) and incubated at room temperature with 100 µL/well of 1:2000 diluted HRP-conjugated anti-human IgG (Becton Dickinson) for two hours. After six additional washing steps, the plates were developed with 100 µL of 0.1% ABTS (Thermo Scientific Fisher Inc., Waltham, MA, USA) solution in ABTS buffer (67.2 mM citric acid, 77.3 mM NaH_2_PO_4_.2H_2_O, and 0.01% H_2_O_2_) and the color development was stopped with 100 µL/well of 0.32% NaF solution after 10 min. Optical densities corresponding to bound antibodies at 405 nm and 492 nm were determined with the Multiskan^TM^ GO microplate spectrophotometer (Thermo Scientific Fisher Inc.) and the difference was plotted (OD_405–492_). For the determination of RBD-specific IgA antibodies, an ELISA was performed with 100 µL of pre-diluted (1:100) serum samples at 4 °C overnight. After six washing cycles, 100 µL of 1:500-diluted anti-human IgA_1_/IgA_2_ mouse IgG_1_ (Beckton Dickinson) was added and the plates were incubated overnight. After six PBS-T washing steps, plates were incubated with 1:1000 diluted HRP-linked anti-mouse IgG_1_ antibody (Cytiva) for 120 min. After six further washing steps, 100 µL/well 0.1% ABTS solution was added for 10 min before stopping the reaction with an equal volume of 0.32% NaF. Finally, absorbances were determined as OD_405–492_. All determinations were carried out in duplicates, blank-subtracted (dilution buffer control), and mean ODs were calculated and normalized according to a reference sample that had been incorporated on each plate. To omit any further variations, the reference sample was performed on a single plate coated with RBD-Wuhan-Hu-1, -Delta, and -Omicron in distinct wells, and the resulting difference, if any, was used to correct the respective OD values of samples to ensure accurate comparisons of specific antibody levels being directed against the different RBD variants. ELISA threshold values were defined as one SD of the mean OD value obtained with the dilution buffer control on the RBD-coated wells (i.e., for IgG; Log_10_: RBD-Wuhan-Hu-1 = −1.93, RBD-Delta = −2.55, RBD-Omicron = −2.78, and for IgA; Log_10_: RBD-Wuhan-Hu-1 = −2.98, RBD-Delta = −2.98, RBD-Omicron = −2.83).

### 2.6. FCCA Adsorption Experiments

Cultured HEK-293T-p cells and RBD-Wuhan-Hu-1-, RBD-Omicron BA.1- as well as Spike-Wuhan-Hu-1-expressing cell lines were harvested 72 h after seeding and washed twice with PBS. Aliquots of 200 µL of serum samples (Table S4), diluted 1:400 in FACS buffer, were incubated with 3 × 10^6^ pelleted cells (2500× *g* for 1 min) of the above-mentioned cell lines, as adsorption matrices, in 1.5 mL screw-cap microtubes (Sarstedt) at 4 °C with continuous agitation using a POLYMAX 2040 shaker (Heidolph Instruments GmbH, Schwabach, Germany) for 1 h. After centrifugation, the supernatant was again incubated with a fresh aliquot of 3 × 10^6^ pelleted cells of the indicated adsorption cell lines in fresh 1.5 mL microtubes. In total, the adsorption procedure was repeated three times, which confirmed the complete adsorption of antibodies during the establishment of the assay procedure. Finally, the adsorbed serum sample was used to stain the abovementioned target cell lines, and the percent decrease in ∆gMFI was calculated in relation to the pre-adsorbed serum sample from the same sample dilution mixture determined against the respective target cell line, according to the formula: $1-\frac{{log10∆gMFI}_{post-adsorbed}-3.14}{{log10∆gMFI}_{pre-adsorbed}-3.14}\times 100\%$.

### 2.7. Western Blot Analyses

Whole-cell lysates of RBD-expressing HEK-293T cell lines and parental HEK-293T cells were obtained by incubating cells harvested 72 h after seeding using 1× PBS at a ratio of 1 × 10^7^ cells in 50 µL lysis buffer containing 1% Triton X-100, 1 mM EDTA, 1 mM PMSF, and 1.5 µM Aprotinin (Roche Diagnostics GmbH, Mannheim, Germany) in MBS buffer (25 mM Mes, 150 mM NaCl, pH = 6.5) on ice for 30 min. Subsequently, lysates were homogenized with 10 gentle strokes of an ice-cold loose-fitting 2 mL Wheaton Douncer (Fisher Scientific, Hampton, NH, USA), followed by centrifugation at 1000× *g* to remove insoluble material at 4 °C for 1 min. The resulting supernatant was aliquoted and stored at −80 °C. For Bis-Tris SDS-PAGE, aliquots of lysates were thawed on ice and volumes corresponding to 5 × 10^5^ cells each were mixed with 4× LDS sample buffer (GenScript Biotech) and incubated at room temperature for 20 min. Positive controls consisting of 50 ng/well of recombinant RBD-Wuhan-Hu-1-Avi-His tag (GenScript Biotech) and RBD-Omicron BA.1-His tag (Sino Biological), also dissolved in 4× LDS sample buffer, were included in the experiments. Samples were prepared by adding distilled water up to 40 µL and were resolved by 4–20% SurePAGE™ (GenScript Biotech) in Tris-MOPS-SDS running buffer (GenScript Biotech). Proteins were transferred onto a nitrocellulose membrane [31] (Bio-Rad) and blocked with 5% skimmed milk (Maresi GmbH, Vienna, Austria) in TBS-T (0.1% Tween-20) at room temperature for 2 h. This was followed by incubation with a 1:1000 diluted anti-SARS-CoV-2 human serum pool (*n* = 5), consisting of highly RBD-reactive sera from the study, in 1% skimmed milk in TBS-T at 4 °C overnight. The next day, bound antibodies were detected using a 1:8000 diluted HRP-conjugated anti-human pan-IgG antibody (Beckton Dickinson) in 1% skimmed milk in TBS-T at room temperature for 45 min. Chemiluminescence was induced with the Clarity Western ECL substrate (Bio-Rad) and detected by imaging (LAS-4000, GE Healthcare, Chicago, IL, USA). To verify uniform sample loading, the nitrocellulose membrane was gently stripped twice in a stripping buffer (0.1% SDS, 1% Tween-20, 1.5% glycine, pH = 2.2) for 30 min and blocked as before. The membrane was re-probed with 1:2000 diluted rabbit anti-human calnexin antibody (Cell Signaling Technology Europe B.V., Leiden, The Netherlands) in 1% skimmed milk in TBS-T at 4 °C overnight. Subsequently, the membrane was incubated with 1:8000 diluted HRP-linked goat anti-rabbit polyclonal Ig (Agilent BioTeK) in 1% skimmed milk in TBS-T at room temperature for 45 min. Chemiluminescence detection of bound antibodies was carried out as mentioned above.

### 2.8. Data and Statistical Analyses

All graphical representations of data were carried out with GraphPad Prism 9.0 (GraphPad Software Inc., La Jolla, CA, USA). To analyze groups with similar variance, either a Kruskal–Wallis test or a Friedman’s test followed by Dunn’s multiple comparisons post hoc testing was applied. For statistical and graphing purposes, ELISA absorbance values of < 0.0001 were manually assigned a value of 0.0001, and ∆gMFI IgG and IgA values of < 200 were assigned a value of 200 in the case of the FCCA. n.s., not significant; *, *p* < 0.05; **, *p* < 0.01; ***, *p* < 0.001.

## 3. Results

### 3.1. HEK-293T Transfectants Expressing GPI-Anchored RBD of SARS-CoV-2 Variants Bind Human ACE2 and Anti-SARS-CoV-2 Antibodies

Antibodies directed against the conformational epitopes of the receptor-binding domain (RBD) of SARS-CoV-2 have been shown to be highly important for inhibiting virus infection by blocking the ACE2–RBD interaction and thus inhibiting virus entry [16,32,33,34]. Moreover, it has been demonstrated that mainly RBD-specific antibodies highly correlate with virus neutralization [35,36,37].

**Figure 1:**
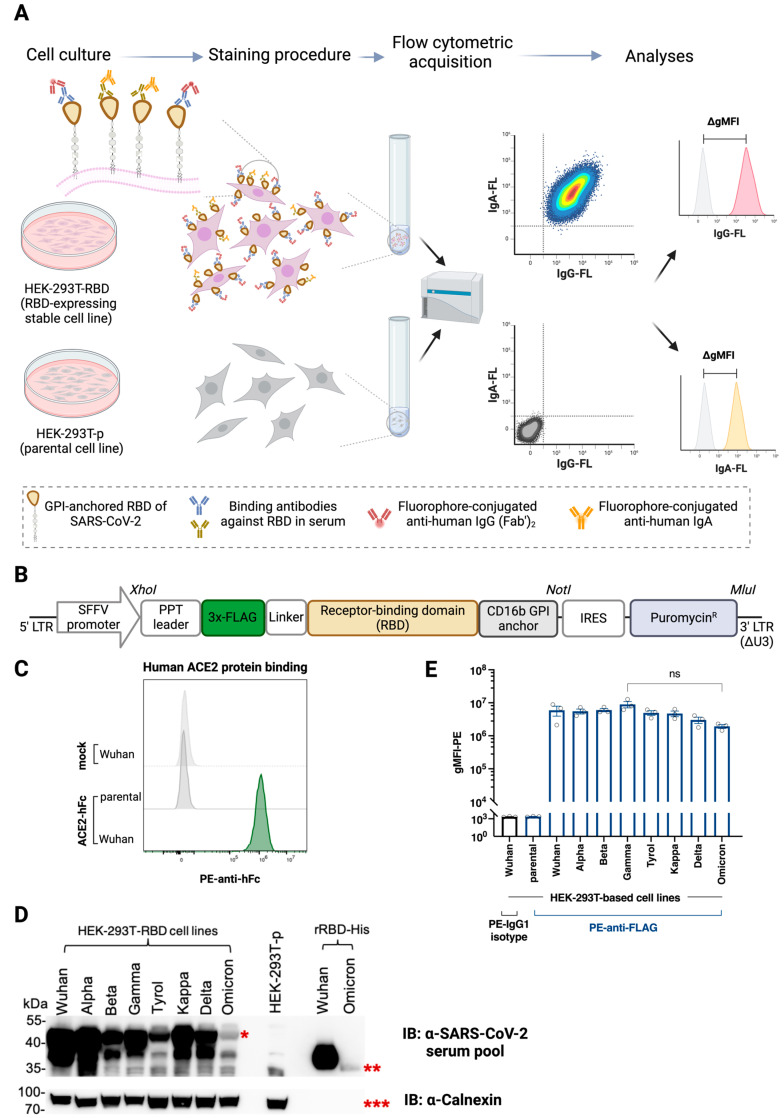
Principle of the FCCA platform and confirmation of folded RBD expression on cell lines. (**A**) Shown is the workflow of the FCCA. HEK-293T transfectants or parental HEK-293T(-p) cells are grown in cell culture, harvested, and subjected to incubation with serum samples. During the staining procedure with the respective serum samples, anti-RBD antibodies will bind to the GPI-anchored RBD proteins displayed on the cell surface, followed by their detection with fluorophore-conjugated anti-IgG or -IgA secondary antibodies, respectively. Cells are then acquired on a flow cytometer and the change in the geometric mean fluorescence intensity (∆gMFI) is calculated as the difference between the signal intensity obtained with the RBD-expressing HEK-293T cell line and the HEK-293T-p cells. (**B**) Representation of the PPT::FLAG::RBD::CD16b-GPI::IRES::Puro expression construct within the pHR-based lentiviral transfer plasmid. (**C**) Stacked histograms show the flow cytometry-based analysis of human ACE2-hFc protein binding to the Wuhan-Hu-1-RBD-expressing HEK-293T cell line (green) in comparison to the non-transduced HEK-293T-p cells (dark grey). (**D**) Shown are the representative Western blot analyses (*n* = 3) of the collection of RBD-expressing HEK-293T cell lysates probed with (upper panel) 1:1000 diluted anti-SARS-CoV-2 human serum pool (*n* = 5), obtained from vaccinated and/or convalescent individuals with high anti-RBD reactivities. Subsequently, the blot was stripped and probed with rabbit anti-calnexin mAb. The protein ladder in kDa is indicated on the left. *, RBD-GPI proteins within cell lysates (~40 kDa); **, glycosylated recombinant RBD from Wuhan-Hu-1 (~35 kDa) and Omicron (~33 kDa); ***, calnexin (90 kDa). Empty lanes were left on either side of the HEK-293T-p lysate. (**E**) Bar graphs show the expression (mean ± SEM) of GPI-anchored FLAG-tagged SARS-CoV-2 variants on stably transfected HEK-293T cell lines (*n* = 3 (circles), each performed in duplicates) determined by flow cytometric analysis. n.s., not significant, as determined by Kruskal–Wallis test followed by Dunn’s multiple comparisons.

We developed a test system for measuring immune responses against functional cell surface-expressed RBD and provided examples of the usefulness of this model for measuring specific immunity against RBD-expressing cells (Figure 1A). Stable expression of RBD was achieved by lentiviral transduction of HEK-293T cells with 3×-FLAG-tagged and PPT leader-driven versions of RBD, linked to the minimal CD16b GPI anchor acceptor sequence for efficient cell surface expression (Figure 1B) [23]. The translation product also contained an IRES-driven puromycin resistance gene for convenient antibiotic selection of transfectants and generation of single-cell clones stably expressing the respective RBD version (Figure 1B). Our study exemplifies that RBD-expressing cell lines can be used to detect human IgG and IgA antibodies present in sera from COVID-19 convalescent patients and SARS-CoV-2 vaccinated individuals by multicolor flow cytometry (Figure 1A, right panel).

The specific binding of recombinant human ACE2-Fc fusion protein to RBD-Wuhan-Hu-1 transfectant but not to parental HEK-293T cells confirmed the proper folding of the surface-expressed RBD (Figure 1C).

Figure 1D shows the immunoreactivity of a serum pool of convalescent and/or vaccinated SARS-CoV-2 individuals (*n* = 5) with the predicted 40 kDa FLAG::RBD::CD16b-GPI protein versions in cell lysates of HEK-293T transfectant but not in parental HEK-293T lysates and reactivity with the ~35 kDa and ~33 kDa proteins representing purified recombinant Wuhan-Hu-1 RBD and Omicron RBD proteins, respectively. The anti-calnexin immunoblot confirmed even sample loading across all cell lysates, including parental HEK-293T cells, revealing a homogeneous protein signal at 90 kDa (Figure 1D, lower panel).

Anti-FLAG staining of the single-cell clones revealed highly specific expression of the eight different RBD versions ranging from Wuhan-Hu-1 to Omicron (Table S3), while no such expression was observed on parental HEK-293T cells or isotype-stained RBD-Wuhan-Hu-1-transfected HEK-293T cells (Figure 1E, Table S3). Thus, FLAG-tag staining can be used to ensure that similar levels of RBD versions were expressed by the different single-cell clones and to calibrate assays performed with cells expressing different RBD versions. ACE2 binding was stable when different batches of the same single-cell clone were tested. The above results assured us that RBD transfectants of HEK-293T cells can be used for the meaningful determination of anti-SARS-CoV-2 antibody binding.

### 3.2. Detection of SARS-CoV-2 Anti-RBD Antibodies Using the RBD Transfectants Revealed That the FCCA Has a Wide Linear Performance Range

**Figure 2:**
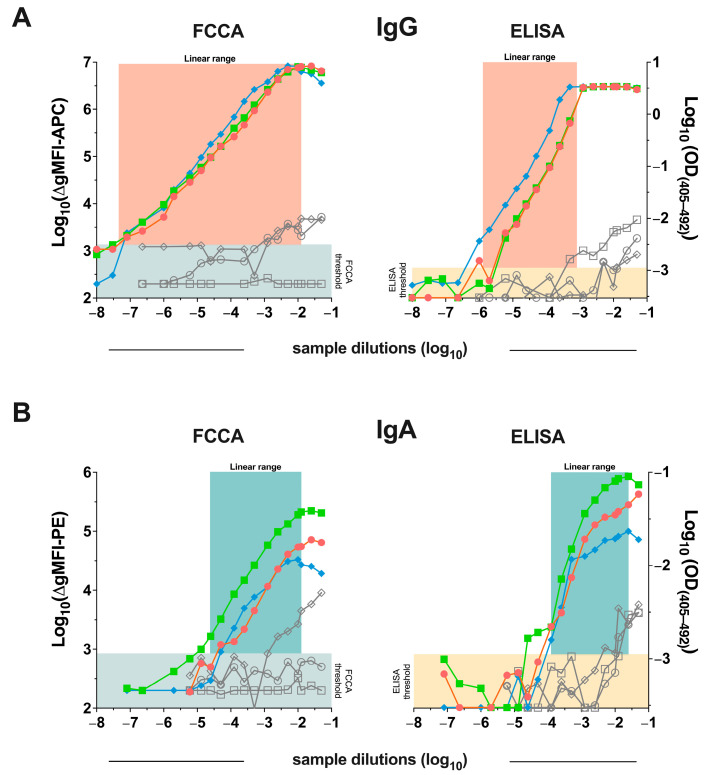
Wide linear dynamic range of the FCCA. Detection of anti-RBD (**A**) IgG and (**B**) IgA antibodies over the indicated large range of serum dilutions from three SARS-CoV-2 exposed and vaccinated (N3-E1, B172-V2, B157-V2; red, green, blue line graphs, respectively) and three SARS-CoV-2 non-exposed (A008, A035, A045; diamond, circle, square shapes in grey, respectively) individuals was performed with the FCCA using HEK-293T-RBD-Wuhan-Hu-1 cells (left). The right parts of the figure show results from an ELISA performed with recombinant RBD-Wuhan-Hu-1. In both (**A**) and (**B**), log_10_-transformed ∆gMFI (left y-axes) values obtained by FCCA and OD_(405–492)_ (right y-axes) acquired in ELISA are displayed. On the x-axes, sample dilutions in log_10_ steps are represented. The estimated linear range window of both assays is highlighted by colored underlays (IgG::red; IgA::cyan). The indicated threshold for FCCA was set at 1 SD of the mean gMFI obtained with the respective secondary antibody (APC-anti-human IgG, PE-anti-human IgA) without serum in 18 independent experiments (IgG::log_10_ = 3.14; IgA::log_10_ = 2.93; light blue color underlay), while the ELISA threshold was fixed at 1 SD of the mean OD value obtained with the dilution buffer control on the RBD-coated wells (i.e., for IgG and IgA; Log_10_: = −2.96; ocher color underlay).

Figure 2A exemplifies the IgG serum reactivity of three convalescent and vaccinated patients and three SARS-CoV-2 non-exposed subjects with the Wuhan-Hu-1-RBD-transfected HEK-293T cells over a wide range of serum dilutions. Results for IgA serum reactivity are shown in Figure 2B. The obtained data demonstrated the wide dynamic range of the FCCA, i.e., > 5 log_10_ steps for IgG (Figure 2A) and > 2.5 log_10_ steps for IgA (Figure 2B). Notably, sera were diluted in PBS in the presence of 0.5–1.0% bovine serum albumin for both the ELISA and the FCCA. Test systems with a high dynamic performance range, such as the herein-described FCCA, may facilitate the evaluation of analytes at a given (single) dilution. Accordingly, the FCCA will be useful for endpoint titration experiments and suitable dilutions can then be selected for comparing different samples.

### 3.3. HEK-293T Cells Expressing Surface-Anchored SARS-CoV-2 RBD Versions Are Suitable for Comparing Specific Antibody Levels in Convalescent and/or Vaccinated Individuals

**Figure 3:**
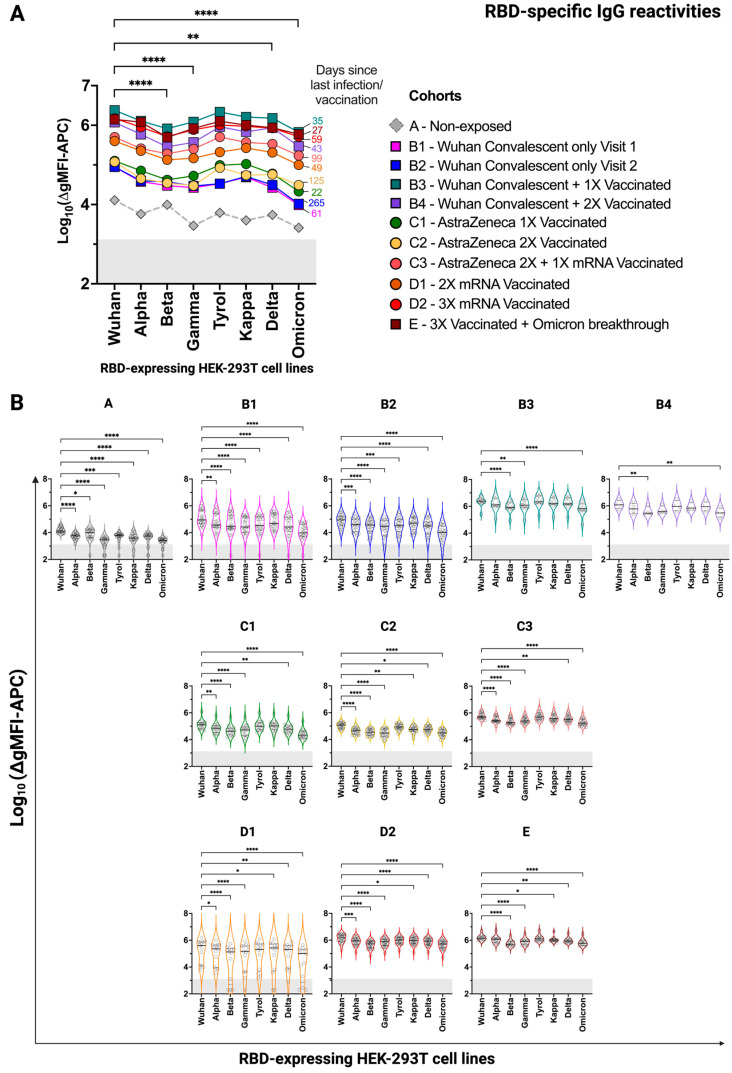
SARS-CoV-2 RBD-specific IgG reactivity of groups of individuals with the indicated infection/vaccination record determined by the FCCA. (**A**) The variant-wise comparison of median SARS-CoV-2 anti-RBD IgG antibodies (1:100) across 11 study groups, including results from a non-exposed group. Squares represent convalescent and/or vaccinated groups while circles show exclusively vaccinated groups. The median time, in days, since the last event (infection/vaccination) is displayed on the right side of each graph in the color of the indicated group. (**B**) The above information represented in group-wise violin plots in the color of the indicated group. Horizontal black lines indicate the median while dotted lines show the upper and lower quartiles. IgG reactivities are displayed as log_10_(∆gMFI-APC). The area indicating the FCCA threshold is shaded in grey, denoting negative values. *, *p* < 0.05; **, *p* < 0.01; ***, *p* < 0.001; ****, *p* < 0.0001 as determined by the Friedman test with Dunn’s multiple comparison correction. In (**A**), the statistical analysis is performed without the non-exposed group.

**Figure 4:**
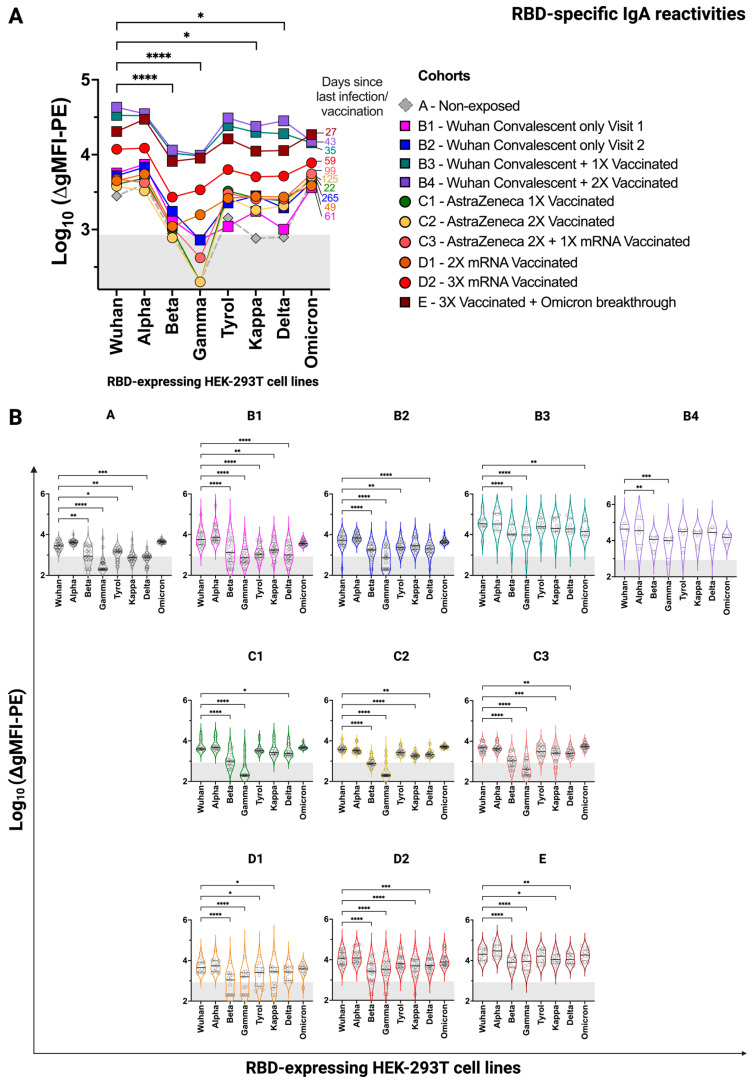
SARS-CoV-2 RBD-specific IgA reactivity of groups of individuals with the indicated infection/vaccination record determined by the FCCA. (**A**) The variant-wise comparison of median SARS-CoV-2 anti-RBD IgA antibodies (1:100) across 11 study cohorts, including a non-exposed group. Squares represent convalescent and/or vaccinated groups while circles show exclusively vaccinated groups. The median time, in days, since the last infection ± vaccination is indicated on the right of each graph in the color of the indicated group. (**B**) The above information represented in cohort-wise violin plots in the color of the indicated group. Horizontal black lines indicate the median while dotted lines show the upper and lower quartiles. IgA reactivities are displayed as log_10_(∆gMFI-PE). Area indicating FCCA threshold is shaded in grey, denoting negative values. *, *p* < 0.05; **, *p* < 0.01; ***, *p* < 0.001; ****, *p* < 0.0001 as determined by the Friedman test with Dunn’s multiple comparison correction. In (**A**), the statistic is performed without the non-exposed group.

In order to study antibody responses to cell lines expressing RBDs from different SARS-CoV-2 variants, groups of convalescent and/or vaccinated individuals were formed, as shown in Table 1, and their serum reactivity was compared along with the serum reactivity of 25 individuals without documented SARS-CoV-2 infection. The evidence for ‘non-exposure’ was purely based on the combination of anamnesis (no symptoms), no positive PCR test on the day of blood sampling, and negative antibody tests. Figure 3A,B show IgG reactivities of the different groups to cell surface-expressed RBDs from Wuhan, Alpha, Beta, Gamma, Tyrol, Kappa, Delta, and Omicron strains. Median IgG levels in Figure 3A indicate that IgG levels to Beta, Gamma, and Omicron were lower than those directed to the other strains in SARS-CoV-2-exposed individuals. Of note, RBD-specific antibody levels were highest in the groups containing convalescent and vaccinated subjects (i.e., groups B3 and B4), three-fold vaccinated subjects (i.e., groups C3 and D2), and in those who received three vaccinations and had experienced an Omicron breakthrough infection (i.e., group E) (Figure 3A,B). RBD-specific IgG antibody levels were considerably lower in only convalescent individuals (i.e., groups B1 and B2) and individuals who had received one or two vaccinations (i.e., groups C1, C2, and D1). RBD-specific IgG antibody reactivities above the cut-off (i.e., the reactivity of the respective secondary-only antibody, plus one standard deviation, with all eight RBD transfectants and the parental HEK-293T cells in 18 independent experiments) were noted in some of the subjects who had not reported SARS-CoV-2 infection (group A) (Figure 3A,B). In principle, sera of previously infected individuals had significantly stronger reactivity with the different RBD transfectants compared to sera derived from SARS-CoV-2 non-exposed individuals. With some exceptions, results were similar for RBD-specific IgA reactivities (Figure 4A,B). The median IgA reactivities for RBDs from Beta and Gamma were especially low whereas the median Omicron-specific IgA levels were quite comparable among the groups. Of note, RBD-specific IgA levels were highest for subjects who were either convalescent and then vaccinated (i.e., groups B3 and B4) or who were vaccinated and then had a breakthrough infection (i.e., group E). That natural infection is a good IgA inducer was also reflected by the much higher correlation coefficient between IgG and IgA levels for infected ± vaccinated individuals compared to only vaccinated individuals (ρ = 0.85 versus ρ = 0.62, respectively, *p* < 0.0001 for both) (Figure S3). RBD-specific IgA levels were lower in only convalescent (i.e., groups B1 and B2) and only vaccinated subjects (i.e., groups C1, C2, C3, D1, and D2) (Figure 4A,B). Again, RBD-specific IgA reactivity above the threshold was found in subjects who had not reported a SARS-CoV-2 infection or vaccination.

For a subgroup of seven patients who were immunized and venipunctured as a cohort, the durability of the anti-RBD IgG levels post-vaccination and as determined by the herein-established FCCA could also be proven. The non-infected individuals (22–37 years of age), who received two shots of AstraZeneca and one shot of a mRNA vaccine, reacted at all three time points most strongly with the RBD-Wuhan-Hu-1-, followed by the RBD-Delta-, and finally with the RBD-Omicron-HEK-293T cell line, as determined after the indicated time points post-vaccination (Figure S4). Especially, the mRNA booster led to a considerable increase in anti-RBD levels that persisted at elevated levels even after 4 months (median) against all three RBD transfectants compared to the individuals who received only two doses of AstraZeneca vaccine, as also shown by others [38].

### 3.4. Excellent Correlation of the FCCA with the ELISA Results

**Figure 5:**
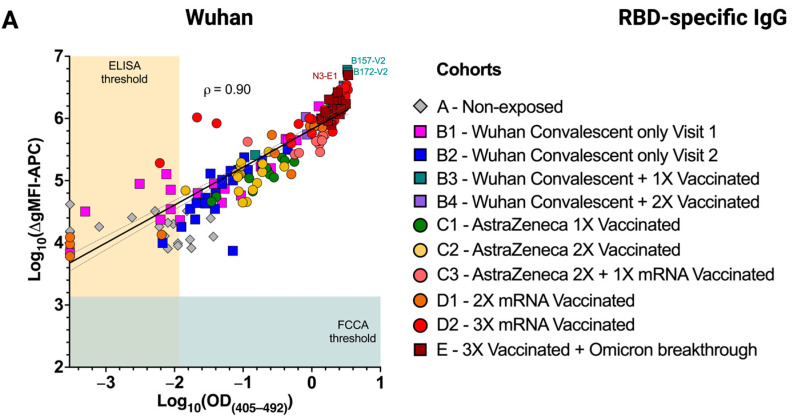

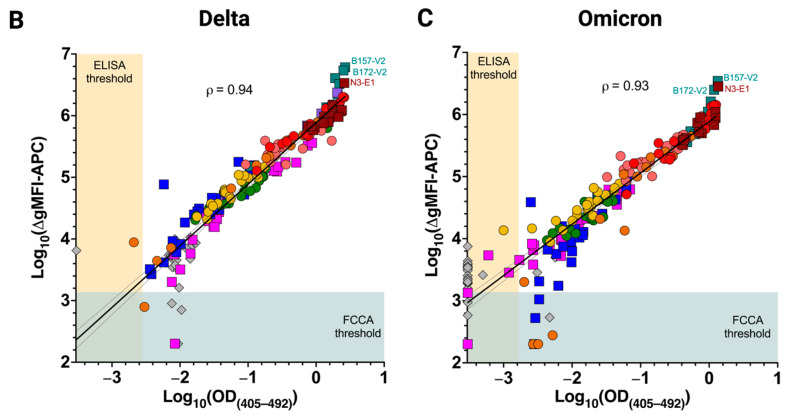
Immunoreactivity of RBD-specific IgG determined by the FCCA correlates with results obtained by ELISA. Scatter plots show a high correlation of anti-RBD IgG levels determined by the FCCA and ELISA against (**A**) RBD-Wuhan-Hu-1, (**B**) RBD-Delta, and (**C**) RBD-Omicron. Each symbol corresponds to a distinct sample and its color depicts the sample group to which it belongs. Sera were diluted 1:100 for the FCCA and 1:200 for the ELISA, respectively. The x-axes show log_10_-transformed OD_(405–492)_ values obtained by ELISA, while the y-axes represent log_10_-transformed ∆gMFI-APC values acquired with the FCCA. The horizontal blue color underlay represents the FCCA threshold, while the vertical ocher color underlay represents the ELISA threshold. Pearson’s correlation, ρ, and the linear regression line with 95% confidence intervals (dotted) are indicated in each graph.

**Figure 6:**
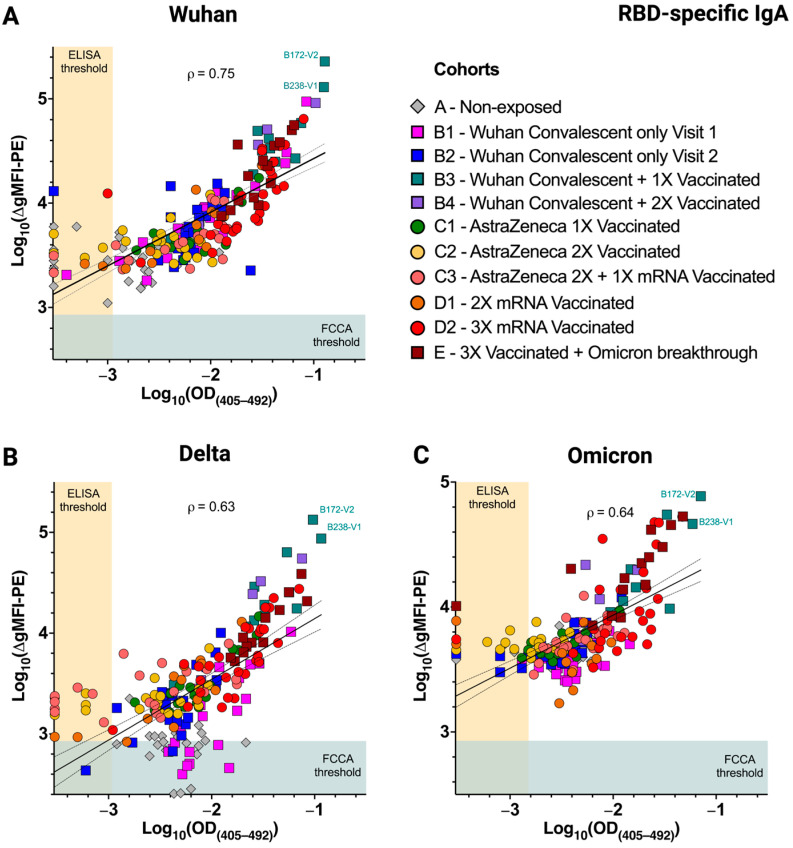
Immunoreactivity of RBD-specific IgA determined by the FCCA correlates with results obtained by ELISA. Scatter plots show a high correlation of anti-RBD IgA levels determined by the FCCA and ELISA against (**A**) RBD-Wuhan-Hu-1, (**B**) RBD-Delta, and (**C**) RBD-Omicron. Each symbol corresponds to a distinct sample and its color depicts the sample group to which it belongs. Sera were diluted 1:100 in both assay formats. The x-axes show log_10_-transformed OD_(405–492)_ values obtained in ELISA while the y-axes represent log_10_-transformed ∆gMFI-PE values acquired with the FCCA. The horizontal blue color underlay represents the FCCA threshold while the vertical ocher color underlay represents the ELISA threshold. Pearson’s correlation, ρ, and the linear regression line with 95% confidence interval (dotted) are indicated on each graph.

Next, we sought to investigate whether the serum RBD reactivities as determined with the transfectant-based FCCA also correlated with those obtained by ELISA. Comparison of the IgG reactivities with the plate-bound RBDs of Wuhan-Hu-1, Delta, and Omicron showed that the immunoreactivity of sera with IgG-specific antibodies correlated extremely well between the two assay systems (ρ = 0.90, 0.94, and 0.93, respectively) (Figure 5A–C). Similar, albeit less well-pronounced, correlations were obtained when the IgA serum reactivities were compared between the two assays (Figure 6) (ρ = 0.75, 0.63, and 0.64 with RBD-Wuhan-Hu-1, -Delta and -Omicron, respectively). Notably, the weakly and strongly reactive sera showed differential binding in the FCCA system while they plateaued in ELISA, which demonstrated the wide linear range of the FCCA and thus its practicability. It may also be assumed, however, that the utilization of different detection systems i.e., fluorophore-conjugated detection antibodies in FCCA versus HRP-linked detection antibodies in ELISA, or, alternatively, the slightly different serum dilution used in the two test systems (FCCA, 1:100 for IgG and IgA; ELISA, 1:200 for IgG and 1:100 for IgA) may account for the observed differences (Figure S6).

### 3.5. Differences in Infection- and Vaccine-Induced Correlations between RBD-Wuhan-Hu-1 IgG and IgA Reactivity

**Figure 7:**
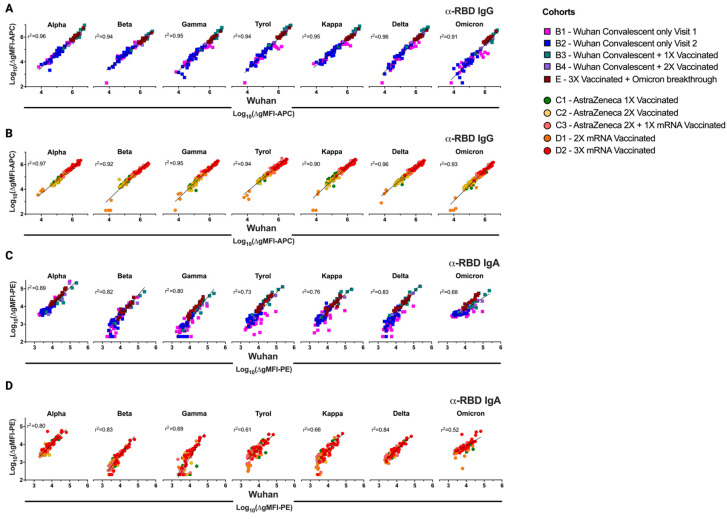
Antibody recognition of the original SARS-CoV-2 RBD predicts binding efficacy to the evolved variants. Predictive correlation analyses of (**A**,**B**) anti-RBD IgG and (**C**,**D**) anti-RBD IgA antibodies against distinct RBD variants of SARS-CoV-2, as determined by the FCCA, grouped according to the convalescent ± vaccinated cohorts (**A**,**C**), and vaccinated-only cohorts (**B**,**D**). RBD reactivity of antibodies against the original Wuhan-Hu-1 strain was used as the predictor on the x-axes. Each symbol corresponds to a distinct sample. Coefficient of determination, *r*^2^, and the linear regression line (black) are indicated on each graph.

Interestingly, the IgG reactivity with RBD-Wuhan-Hu-1 observed with the sera of the ten differently exposed groups clearly correlated (*r*^2^ > 0.91, *p* < 0.0001 for all analyses) with the reactivity of the same sera with the RBDs of the seven other major variants tested herein (Figure 7A), which was in accordance with the findings of others [39]. The strong correlations were independent of whether the individuals were only infected, only vaccinated, or both infected and vaccinated (Figure 7A,B). However, with regards to IgA reactivity, a different picture was observed. While the Wuhan-Hu-1-RBD-specific IgA levels of individuals who were infected and/or vaccinated predicted the IgA reactivity also to the RBDs of the other VOCs very well (groups B1–B4 and E, *r*^2^ range 0.68–0.89, *p* < 0.0001 for all) (Figure 7C), a much weaker correlation was observed for the sera of groups C1-C3 and D1–D2, i.e., those derived from patients who were solely vaccinated (*r*^2^ range 0.52–0.84, *p* < 0.0001 for all) (Figure 7D). This may indicate that IgA-producing B cells/plasma cells originating from infection may be of a different clonal origin than IgG-producing B cells/plasma cells induced by vaccination. This finding may have important implications for the future induction of SARS-CoV-2 IgA-driven mucosal immunity by vaccines.

### 3.6. Excellent Sensitivity and Specificity of the FCCA Underscores Its Usefulness as a SARS-CoV-2-Specific Diagnostic Test

**Figure 8:**
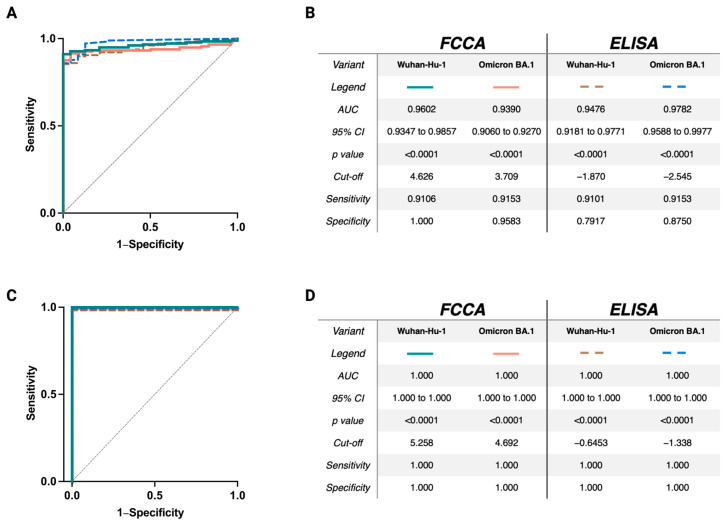
Receiver operating characteristic (ROC) curves confirm the FCCA as a highly specific and highly sensitive assay for the determination of anti-RBD antibodies. Shown in (**A**,**C**) are the ROC curves of the FCCA (orange and green lines) and ELISA (brown and blue dotted lines) obtained by comparing the IgG reactivity against RBD-Wuhan (green and brown) and RBD-Omicron (orange and blue) of the non-exposed individuals (group A; *n* = 24) to (**A**) all convalescent and/or vaccinated individuals included in the study (groups B1-E; *n* = 179) and (**C**) 3X-vaccinated plus Omicron convalescent individuals (group E; *n* = 14). Grey diagonal lines show random classifiers. The tables in (**B**,**D**) show the area under the curve (AUC), 95% confidence intervals, *p* values, cut-offs for the determination of seropositivity, and the respective sensitivity and specificity.

To determine the sensitivity and specificity of the FCCA for detecting anti-RBD IgG antibodies in human serum samples, receiver operating characteristic (ROC) analyses were conducted (Figure 8). When comparing the serum reactivities of all non-SARS-CoV-2-exposed subjects (group A; *n* = 24) against all SARS-CoV-2-exposed subjects (by infection and/or vaccination; groups B1–E; *n* = 179), the FCCA had an excellent sensitivity of 91% and a specificity of 96% (Figure 8A,B). ROC evaluations showed that the FCCA compared to the ELISA, assuming the same true positive rate (sensitivity), performed highly comparably if not better than the ELISA to identify true negatives (specificity). As IgG antibodies diminish over time, both in convalescent and vaccinated individuals, ROC curves were also generated by comparing the serum reactivity of individuals of group E (3X vaccinated plus breakthrough infection; *n* = 14) with a median phlebotomy time point of 27 days from the breakthrough infection (Table 1) with group A (non-exposed healthy controls). This comparison revealed a sensitivity and specificity of 100%, respectively (Figure 8C,D).

### 3.7. The FCCA Platform May Identify SARS-CoV-2-Specific Antibodies in Individuals without Evidence of a SARS-CoV-2 Infection

**Figure 9:**
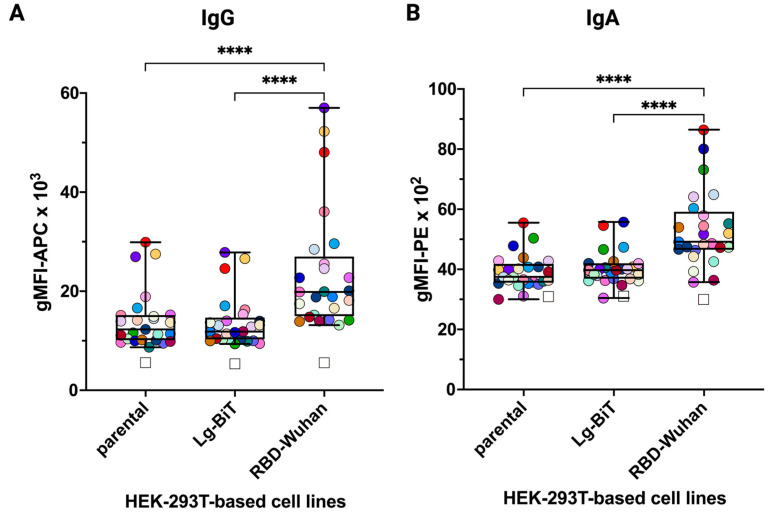

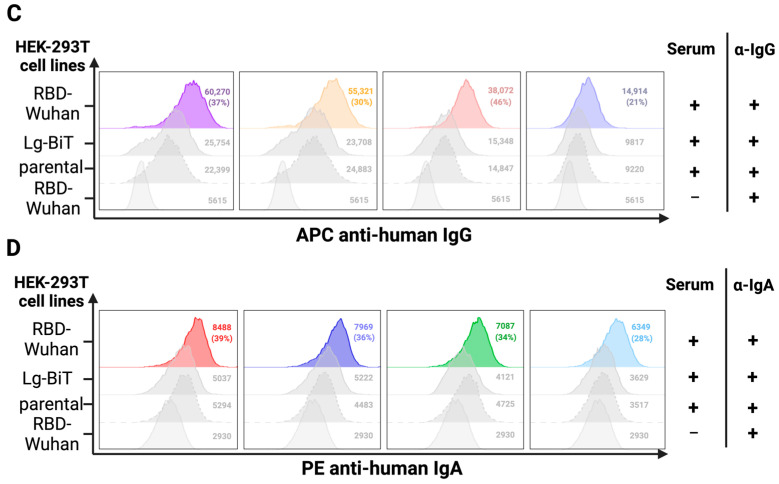
The FCCA may detect low levels of pre-existing cross-reactive anti-RBD antibodies in non-SARS-CoV-2-exposed individuals (Group A). Shown in (**A**,**B**) are pre-existing IgG and IgA antibodies binding to the SARS-CoV-2 RBD-Wuhan-Hu-1 in the SARS-CoV-2 non-exposed group A of individuals (*n* = 25). Y-axes show raw gMFI values. The reactivities with the parental HEK-293T cell line as well as against the transduction control HEK-293T-Lg-BiT stable cell line are shown as controls. Each dot represents the reactivity of a single serum sample (dilution 1:100) with the three different cell lines (parental, Lg-BIT, and RBD-Wuhan-Hu-1). The open squares show the reactivity (gMFI) of the respective secondary antibodies with the three different cell lines in the absence of prior incubation with serum. In the plot, the boxes indicate the upper and lower quartiles, with the horizontal line showing the median, with whiskers representing minimum and maximum values. ****, *p* < 0.0001 as determined by the Friedman test with Dunn’s multiple comparison correction. (**C**,**D**) Stacked histograms of the IgG or IgA reactivity in (**A**,**B**), respectively, of the four sera upon incubation with the RBD-Wuhan-Hu-1 transfectants compared to control transfectants (Lg-BiT) or parental HEK-293T cells (similar color code as in **A**,**B**). Sera were selected on the basis of high and low binding characteristics. In each histogram, the gMFI values are indicated and the percent positive values resulting from the comparison of serum reactivity between RBD-Wuhan-Hu-1 and Lg-BiT control transfectants are shown in parentheses. In the bottom-line histograms of each panel, the reactivity of the secondary antibody alone, in the absence of serum, to RBD-Wuhan-Hu-1 transfectants is shown.

The fact that the FCCA seemed to identify subjects without evidence of a SARS-CoV-2 infection or vaccination prompted us to take a closer look at the reactivity of sera from group A. Figure 9A,B demonstrate the reactivity of group A sera with RBD-Wuhan-Hu-1 transfectants when compared to the parental or pHR_Lg-BiT-transfected HEK-293T cells (i.e., negative controls). In fact, median IgG reactivity amounted to a gMFI of 19,845 with RBD-Wuhan-Hu-1 cells, while it reached gMFI values of only 11,860 and 12,287 with Lg-BiT and parental cells, amounting to ∆gMFI values of 7985 and 7558, respectively. Similarly, the median IgA reactivity of group A sera with RBD-Wuhan-Hu-1 cells, with a gMFI of 4917, was significantly higher than the ones obtained upon incubation with Lg-BiT (gMFI = 3974) and parental (gMFI = 3749) cells, amounting to ∆gMFI values of 943 and 1168, respectively. These differences are appreciable in stacked overlay histograms for both antibody classes, as shown for selected sera (Figure 9C,D). Since all individuals within group A tested negative with a certified SARS-CoV-2 antibody test (Elecsys^®^ Anti-SARS-CoV-2 assay Roche) and had no medical history of infection with SARS-CoV-2, other possibilities than SARS-CoV-2 exposure for the presence of the detected RBD-specific low-level IgG and IgA reactivities may be considered, among them cross-reactivity with other coronaviruses. Interestingly, the baseline reactivity of sera from the non-exposed individuals significantly differed between RBD mutants derived from SARS-CoV-2 variants (Figure 3B and Figure 4B). Also, among the RBD variants, a considerable number of sera had reactivities below the threshold, which highlighted the specificity of the FCCA as an internal control to rule out false positives.

### 3.8. Adsorption Studies with RBD Transfectants Indicate Differences in RBD-Specific Antibody Composition in Vaccinated Individuals with and without Breakthrough Infection

**Figure 10:**
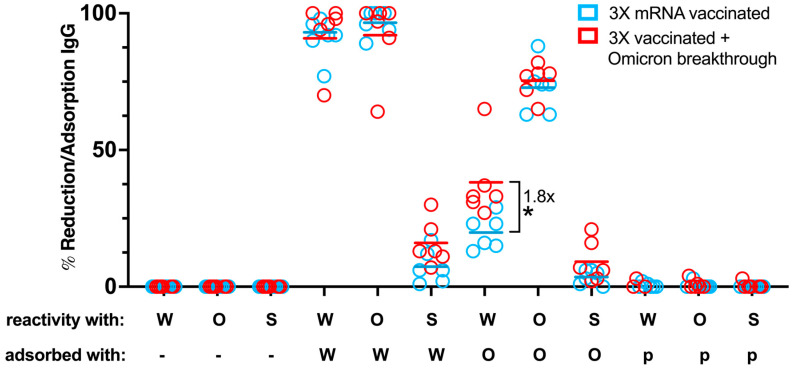
Breadth of SARS-CoV-2 RBD- and Spike-specific IgG repertoire after Omicron breakthrough in 3X vaccinated individuals. The graph illustrates the percentages of adsorbed/reduced specific IgG reactivity determined for sera of two matched cohorts, 3X mRNA vaccinated (blue) and 3X vaccinated + Omicron breakthrough (red), each consisting of six individuals (Table 3), when tested against SARS-CoV-2 RBD-Wuhan-Hu-1 (W), RBD-Omicron BA.1 (O), and Spike-Wuhan-Hu-1 HEK-293T cell lines (S) after different adsorption conditions: (i) non-adsorbed (-), (ii) adsorbed with RBD-Wuhan-Hu-1-HEK-293T cells (W), (iii) adsorbed with RBD-Omicron BA.1-HEK-293T cells (O), (iv) adsorbed with parental HEK-293T cells (p), respectively. Horizontal lines within each column represent mean values. Unpaired two-tailed *t*-test was performed and the only significant difference between the two groups within one condition is indicated. *, *p* < 0.05.

In another approach, we studied whether RBD transfectants could be used for determining the levels of cross-reactive antibodies directed against RBDs of Wuhan-Hu-1 and Omicron BA.1 and the percentage of S-specific IgG antibodies that are directed to RBD-Omicron BA.1. For the adsorption studies, we used sera from six subjects who had received three Wuhan-Hu-1-specific mRNA vaccinations and sera from six individuals who in addition had an Omicron breakthrough infection (Table 3) (Figure 10).

Figure 10 shows that preincubation with the RBD-Wuhan-Hu-1 cell line was able to adsorb 92 ± 9% (mean ± SD) of RBD-Wuhan-Hu-1- and 94 ± 10% of Omicron-specific IgG reactivity from sera of the aforementioned two groups, indicating that the majority of IgG antibodies were directed to the RBD from Wuhan-Hu-1 in these sera.

This would indicate that the breakthrough infection with Omicron induced only low levels of RBD-Omicron-specific IgG. When using sera from patients who had received three mRNA Wuhan vaccinations and had an Omicron breakthrough infection, more IgG could be pre-adsorbed against Wuhan S by RBD from Wuhan and RBD from Omicron than in subjects who had received only three mRNA Wuhan vaccinations. Notably, adsorption with the Omicron RBD showed a significant (*p* = 0.016) 1.8-fold greater reduction of residual RBD-Wuhan-Hu-1 IgG reactivity of sera from subjects with a breakthrough infection as compared to those without a breakthrough infection (38 ± 14% versus 20 ± 6%).

The finding that pre-adsorption with the Omicron RBD yielded a higher reduction of IgG binding in patients with an Omicron breakthrough infection than in those without seemed to suggest that antibodies induced by Omicron infection recognize different epitopes than those recognized by IgG antibodies induced by Wuhan mRNA vaccination. It should also be emphasized that only a small percentage of Wuhan S-specific IgG was directed against the Wuhan RBD in both groups, indicating that mRNA vaccination alone or in combination with an Omicron breakthrough infection induced only a small percentage of RBD-specific IgG antibodies.

## 4. Discussion

Antibodies toward functional and folded RBD from SARS-CoV-2 are important for inhibiting the docking of the virus to its cognate receptor ACE2 on human cells and thus play an important role in virus neutralization [40]. Here, we report the generation and performance of test platforms based on cells stably expressing RBD genes of the eight previously prevalent SARS-CoV-2 strains, ranging from Wuhan-Hu-1 to Omicron BA.1, as FLAG-tagged and CD16b-GPI anchored molecules in human HEK-293T cells. Puromycin-selected single-cell transfectants showed clear-cut and comparable expression of the different RBD versions, as determined by staining for the N-terminally introduced FLAG-tag. Accordingly, the cell lines established by us can be easily standardized regarding their RBD expression by staining with FLAG-tag-specific antibodies. Importantly, the reactivity of transfectants with human ACE2 by multicolor flow cytometry confirmed the proper folding of cell surface-expressed RBD.

We exemplify the usefulness of the cell-based diagnostic platform for SARS-CoV-2 in two applications. In the first set of experiments, we show that RBD-expressing cell lines are useful for the determination of SARS-CoV-2 serum antibody binding. The use of stable RBD-expressing cell lines selectable with puromycin, in which the RBD and the puromycin cDNA are part of a large cistron, enabled consistent expression of RBD by HEK-293T cells [25] and allowed for the subsequent determination of antibody reactivity in a highly reproducible fashion (Figure S7). Furthermore, the FCCA offered a wide linear performance range and improved resolution with high sensitivity and specificity (Figure 2 and Figure 8) for the detection of RBD-specific IgG and IgA antibodies. Of note, there was a high correlation between the FCCA and the ELISA based on plate-bound recombinant RBD proteins. The measurement of RBD-specific IgA antibodies could be very important for local secretions where IgA antibodies form a first line of defense against the virus [41,42]. Interestingly, we found that levels of RBD-specific IgA antibodies were high in subjects who had experienced an infection and who had been vaccinated. Furthermore, among the individuals with an Omicron breakthrough infection (group E, Table 1), the IgA RBD-Omicron reactivity was not significantly different from the IgA RBD-Wuhan reactivity (Figure 4B). A correlation analysis of antibody responses of only vaccinated subjects with those of subjects who had also experienced an infection indeed indicated that IgA-producing B cells/plasma cells originating from infection may be of different clonal origin than IgG-producing B cells/plasma cells induced by vaccination (Figure 7). One, therefore, may consider refining current vaccination technologies regarding better induction of IgA antibodies to harness the first line of mucosal immunity against SARS-CoV-2, and the herein-developed FCCA may be helpful in monitoring vaccination efficacy [43]. Furthermore, the FCCA method seemed to be also very sensitive regarding the detection of RBD-specific antibodies because such antibodies could also be identified in individuals who had no evidence of SARS-CoV-2 exposure, which has been also reported by others [44,45]. These antibodies may originate from previous encounters with endemic *Coronaviridae* or mild and/or silent SARS-CoV-2 infections [46]. However, the significance of the presence of such antibodies in the non-exposed individuals is still controversially discussed [47]. 

In the second application, we demonstrated the usefulness of the FCCA for the adsorption of variant-specific RBD antibodies from whole serum for the subsequent determination of remaining cross-reactive RBD antibody binding, which may be relevant for the monitoring of vaccination success after the recent introduction of bivalent mRNA vaccines. With their surface-expressed RBD variants, the generated HEK-293T-RBD transfectants may also serve the purpose of a novel vaccination platform (for human and/or veterinary use), either composed of the entire membrane fractions [48,49,50] or in the form of subunit vaccines, e.g., based on virus-like nanoparticles (VNPs) (Kratzer et al, manuscript in preparation and [23]) or exosomes [51,52].

Previously, bead- and cellular-based systems for the flow cytometric evaluation of S, RBD, and NC serum reactivity have been established in variable combinations [53,54,55,56,57,58,59,60]. The inherent disadvantage of bead-based systems comes from the fact that the proteins to be loaded onto them have to be expressed, purified, and examined for proper folding in separate protein expression and biochemical experiments, making the generation of antigen-loaded beads a challenge for all those laboratories that are not equipped with biochemistry, protein expression, and purification units. Therefore, flow cytometry systems based on HEK-293T [55,56,59,60] or Jurkat cells [57,58] expressing full-length S-protein have been built. In contrast to the cellular platforms developed previously, our platform is the first to express on HEK-293T cells truncated versions of the SARS-CoV-2 S protein restricted to its RBD domain through a GPI anchor as stably transfected and puromycin-selectable RBD transfectants, which allow to read out the virus-neutralizing antibodies in a highly reproducible manner with low batch-to-batch variations. Furthermore, the introduction of a FLAG tag in our RBD versions provided us with the opportunity to standardize the expression levels of RBD from different variants and to compare both IgG and IgA binding in a semi-quantitative manner. This approach overcomes potential limitations related to the reported affinity differences between the different RBD variants but also Ig isotypes when ACE2-IgG_1_Fc binding is used for standardization [59].

The FCCA-based technology for detecting RBD-specific antibodies has several advantages over solid-phase assays such as ELISA, for instance: (i) determination of the reactivity with a given RBD transfectant and the respective control transfected HEK-293T cell line by incubation with both the serum in question and the fluorescently labeled secondary antibody allows the exact correction (by subtraction) of the background reactivity; (ii) HEK-293T cells can be adapted to novel possible upcoming variants of concern (VOCs) quickly; (iii) the use of the cell lines allows for serological determination of “neutralizing antibody immunity” by concentrating on RBD rather than total S reactivity, the latter being widely used in commonly available tests; (iv) the cell lines can be grown in large numbers and maintained as high expressors due to an IRES-driven puromycin cassette located within the integrated lentiviral pHR expression vector derived sequences and applied with minimal batch-to-batch variation; (v) generation of the immunogen is cost-effective and can be easily scaled up to billions of cells, for instance in Cell Factory systems (Thermo Scientific), suitable for 5–10,000 individual serum evaluations, for those who are operating a standard tissue culture laboratory; (vi) the cellular system is suitable for multiplexing by labeling the respective HEK-293T cell transfectants with differently conjugated antibodies against either the surface-expressed “housekeeping molecules,” e.g., HLA class I, CD59, or the integrins CD29 and CD51, or the adenovirus receptor CD46 [61,62], etc., before mixing and then incubating them with the respective sera to be evaluated—such a strategy may also allow to admix parental or control transfected HEK-293T cells for immediate (one-tube) background correction; (vii) multicolor FCCA allows for determination of more than one (up to all five) specifically binding immunoglobulin classes in a single tube by applying differentially conjugated secondary antibodies and is, in that respect, only limited by the type of flow cytometer used; (viii) our test can be easily benchmarked to available WHO serum standards for subsequent quantification of serum antibodies; (ix) the determination of the serum IgA/IgG-reactivity with all VOCs may inform the personal level of protection; (x) the dual evaluation of specific IgG and IgA may also help to identify high-titer, neutralizing antibody levels in the serum of donors to obtain potentially therapeutic plasma and/or for the generation of humanized blocking antibodies; (xi) use of small quantities of serum samples may facilitate the evaluation of specific antibody binding in low-volume samples, such as bodily secretions; (xii) HEK-293T RBD transfectants can also be used as target cells to determine RBD-specific antibody-dependent cellular cytotoxicity (ADCC) by either patient’s NK cells or even a generic NK cell line (NK-92) in conjunction with patient serum—such assays could be contrasted to assays performed with HEK-293T cells expressing the full-length S-protein to cover all antibodies, also those that are non-neutralizing but still functional in ADCC; and (xiii) pre-incubation of RBD transfectants with individual sera followed by determination of the residual ACE2 binding may help to determine neutralization titers, to name just a few advantages.

This study also has some limitations. For instance, individuals with suspected Wuhan-Hu-1 and Omicron BA.1 breakthrough infections were not directly tested for variant-specific virus RNA by PCR during infection. Instead, the time point of infection was taken as a proxy for attributing the infections to either the Wuhan-Hu-1 or the Omicron variant. Furthermore, the levels of RBD expression need to be controlled and results have to exclude potential confounders caused by cell viability. In addition, it will be necessary to introduce standards based on defined concentrations of RBD-specific antibodies to obtain quantitative results for RBD-specific antibodies. It is also important to indicate that the groupings and the evaluation of the serum reactivities with the transfectants shown herein were not intended to elucidate differences between the different exposure groups. This was not possible since groups of patients were heterogeneous regarding the time points of venipuncture relative to the last (natural or by vaccination) SARS-CoV-2 antigen exposure. Rather, the groupings served to evaluate the basic antigenicity of the transfectants and to highlight possible variant-specific differences in serum reactivities within a group, which could be afforded by testing them against the entire panel of RBD transfectants.

In summary, we have established a simple and highly versatile FCCA-based system for the enumeration of different antibody classes being specific for all eight so far surfacing VOCs of SARS-CoV-2. The novel assay system determines the relevant conformation-dependent antibodies with specificity for RBD variants and can be easily adapted to newly emerging variants within a short period of time. Due to its high sensitivity, the FCCA allows for the evaluation of cross-reactive B cell/antibody memory against SARS-CoV-2. The test principle is not limited to the enumeration of humoral immunity against *Coronaviridae* but may be extended to other important microbial or even tumor antigens.

## Figures and Tables

**Table 1:** Group designation, demographics, and clinical characterization of the study populations.

				Age			Time to Last Event ^#^ (days)		
No.	Group	Type of SARS-CoV-2 Exposure	Individuals (n)	Female (%)	Range	Mean	SD ^*^	Range	Median
**1**	A	Non-exposed	25	16 (64)	19–68	51	13	n.a.	
**2**	B1	Wuhan-Hu-1 Convalescent only (Visit 1)	25	10 (40)	22–76	49	15	37–246	61
**3**	B2	Wuhan-Hu-1 Convalescent only (Visit 2)	25	9 (36)	18–78	49	15	248–306	265
**4**	B3	Wuhan-Hu-1 Convalescent + 1X Vaccinated	11	5 (45)	22–77	49	19	20–89	35
**5**	B4	Wuhan-Hu-1 Convalescent + 2X Vaccinated	4	2 (50)	39–68	49	13	21–103	43
**6**	C1	AstraZeneca 1X Vaccinated	17	13 (76)	22–61	40	14	21–29	22
**7**	C2	AstraZeneca 2X Vaccinated	21	14 (67)	22–59	40	13	96–188	125
**8**	C3	AstraZeneca 2X + 1X mRNA Vaccinated	22	16 (73)	24–69	37	14	23–134	99
**9**	D1	2X mRNA Vaccinated	13	5 (38)	21–85	49	18	21–213	49
**10**	D2	3X mRNA Vaccinated	27	15 (56)	21–68	39	14	20–122	59
**11**	E	3X Vaccinated + Omicron breakthrough	14	7 (50)	23–62	38	13	22–74	27

^*^ Standard deviation; ^#^ Event refers to vaccination or infection; Inclusion criterium was ≥20 days since last exposure by infection or vaccination; No., number; n.a., not applicable.

**Table 2:** Protein sequences of the SARS-CoV-2 RBD-Wuhan-Hu-1 expression construct.

Protein	Amino Acid Sequence ^*^
Pre-pro-trypsin (PPT) leader sequence ^†^	MNPLLILTFVAAALA
FLAG-tag	DYKDHDG*DYKDHDI*DYKDDDDK
Linker sequence	GGGGS
RBD (Wuhan-Hu-1)	PNITNLCPFGEVFNATRFASVYAWNRKRISNCVADYSVLYNSASFSTFKCYGVSPTKL NDLCFTNVYADSFVIRGDEVRQIAPGQTGKIADYNYKLPDDFTGCVIAWNSNNLDS KVGGNYNYLYRLFRKSNLKPFERDISTEIYQAGSTPCNGVEGFNCYFPLQSYGFQPTN GVGYQPYRVVVLSFELLHAPATVCGPKKSTNLVKNKCVNFNFNGLTGTGVLTESNK KFLPFQQFGRDIADTTDAVRDPQTLE
Minimal GPI-anchor acceptor sequence of human CD16b ^#^	SLAVSTISSFSPPGYQVSFCLVMVLLFAVDTGLYFSVKTNI

^*^ Shown is the amino acid sequence of the expression construct; ^†^ PPT leader derived from human serine protease 1 protein sequence (UniProt ID: P07477) [26]; 3×-tandem FLAG tag is represented by bold, italics, and regular font letters; ^#^ After the GPI-anchor acceptor sequence of the SARS-CoV-2 RBD construct, an IRES sequence was placed followed by a puromycin resistance gene [25]; The insert was ligated into the pHR vector by XhoI-NotI restriction enzymes to generate pHR_PPT::FLAG::RBD::CD16b-GPI::IRES::Puro plasmid (Figure S1).

**Table 3:** Characteristics of the groups evaluated in the FCCA adsorption experiments.

				Age			Time to Last Event ^#^ (days)	
Group	Type of SARS-CoV-2 Exposure	Individuals (n)	Female (%)	Range	Mean	SD ^**^	Range	Median
D2 *	3X mRNA Vaccinated	6	3 (50)	23–45	34	7	20–50	29
E *	3X Vaccinated + Omicron breakthrough	6	3 (50)	23–43	32	7	23–47	26

* Sub-groups of the parent cohorts in Table 1; ^**^ Standard deviation; ^#^ Event refers to vaccination or infection; Inclusion criterium was ≥20 days since last exposure by infection/vaccination. Notably, all sera were drawn from the subjects prior to the approval of bivalent-adapted mRNA vaccines by the European Medicines Agency (EMA) and subjects are therefore likely to have received only the monovalent mRNA vaccine.

## Data Availability

The datasets used and/or analyzed during the current study are available from the corresponding author upon reasonable request.

## Supplementary Materials

The following supporting information can be downloaded at: https://www.mdpi.com/article/10.3390/vaccines12040377/s1, Figure S1: Representation of the expression cassette in the pHR vector. Figure S2: Alignment of the amino acid sequences of RBD proteins of SARS-CoV-2 variants. Figure S3: Correlation between IgG and IgA reactivity directed against RBD-Wuhan-Hu-1 as determined with the FCCA using sera from convalescent ± vaccinated or only vaccinated subjects. Figure S4: Time course of the waning of anti-RBD IgG reactivity after vaccinations. Figure S5: Uncropped Western Blot images with densitometry readings. Figure S6: Correlation of IgG to IgA ratios between FCCA and ELISA. Figure S7: Determination of IgG and IgA anti-RBD antibody reactivity using stably expressed RBD on HEK-293T cells by the FCCA is highly reproducible. Figure S8: SARS-CoV-2 RBD-specific IgG reactivity of groups of individuals with the indicated infection/vaccination record determined by the FCCA (multiple comparisons in all cell lines). Figure S9: SARS-CoV-2 RBD-specific IgA reactivity of groups of individuals with the indicated infection/vaccination record determined by the FCCA (multiple comparisons in all cell lines). Table S1: List of primer pairs used for the introduction of point mutations into the SARS-CoV-2 RBD expression constructs. Table S2: List of SARS-CoV-2 RBD expression constructs. Table S3: List of HEK-293T-based single-cell clones stably expressing SARS-CoV-2 RBD variants. Table S4: List of antibodies, staining reagents, and recombinant proteins used in this study.

## Abbreviations

ACE2	angiotensin-converting enzyme 2
ADCC	antibody-dependent cellular cytotoxicity
FCCA	flow cytometry-based cellular assay
gMFI	geometric mean fluorescence intensity
GPI	glycosylphosphatidylinositol
HAT	hemagglutination-based test
IRES	internal ribosome entry site
OD	optical density
PANGO	phylogenetic assignment of named global outbreak lineages
PPT	pre-pro-trypsin
Puro	puromycin resistance gene
RBD	receptor-binding domain
ROC	receiver-operating characteristic
S	spike (SARS-CoV-2 protein)
SCCs	single-cell clones
SD	standard deviation
SI	stimulation index
VOC	variant of concern
VOI	variant of interest
WHO	World Health Organization
