# Supplementary material for: Flow Cytometry-Based Measurement of Antibodies Specific for Cell Surface-Expressed Folded SARS-CoV-2 Receptor-Binding Domains
Source: Vaccines (Basel). 2024 Apr 1;12(4):377. doi: 10.3390/vaccines12040377 (PMC11053794; doi:10.3390/vaccines12040377)
Supplement: Supplementary file 1 [file vaccines-12-00377-s001.zip › vaccines-2928991-supplementary.pdf]

FIGURE S1

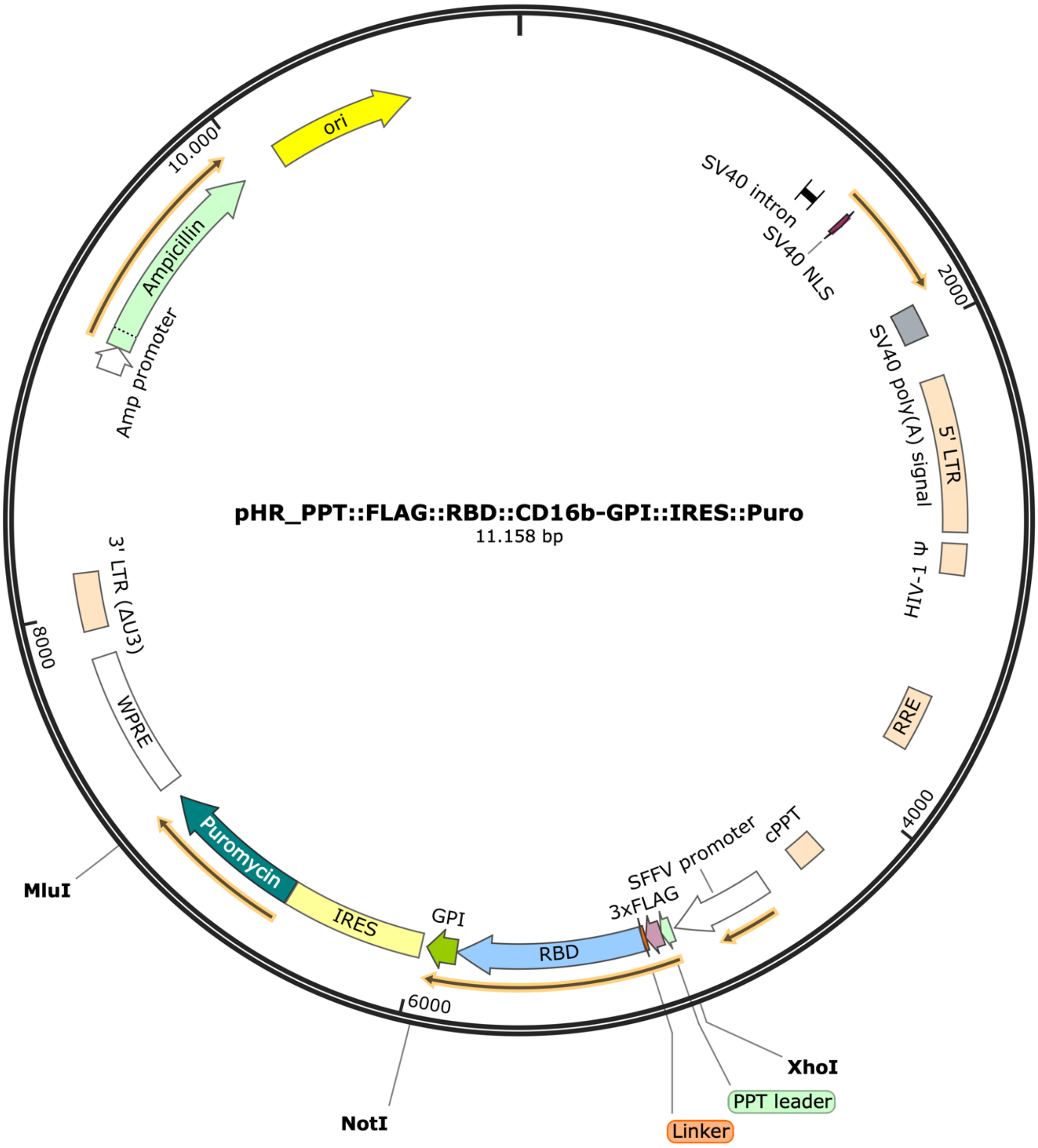

FIGURE S1. Representation of the expression cassette in the pHR vector. The diagram illustrates the composition of the self-inactivating (SIN) pHR_PPT::FLAG::RBD::CD16b-GPI::IRES::Puro transfer plasmid, the map was designed using the SnapGene® software. Upon transduction, the plasmid (size 11,158 bp), a part of HIV (Human immunodeficiency virus)-derived vector system,

integrates into the genome of HEK-293T cells using LTRs (long terminal repeats). Shown are bacterial and eukaryotic antibiotic resistance genes, Ampicillin and Puromycin respectively, the latter is expressed under the influence of SFFV (spleen focus-forming virus) promoter through the IRES (internal ribosome entry site)-mediated translation upon stable transfection. The size of the FLAG::RBD::CD16b-GPI expression cassette is 1022 bp which is cloned into the vector using XhoI-NotI restriction sites (Figure 1) while the IRES::Puromycin DNA sequence was introduced using NotI-MluI restriction sites. PPT-leader indicates pre-pro-trypsin leader sequence derived from human serine protease 1 protein sequence (UniProt ID: P07477), linker encodes GGGGS, CD16b-GPI acceptor sequence anchors RBD on the surface of the cell membrane. Ori, origin of replication; SV40 (simian virus 40) intron; SV40 polyadenylation signal; SV40 NLS (nuclear localization signal); psi (Ψ), RNA packaging site; RRE, rev response element; cPPT, central polypurine tract; WPRE, Woodchuck hepatitis virus post-transcriptional regulatory element; ΔU3, deleted unique 3′ are shown.

FIGURE S2

```
Wuhan-Hu-1 330 PNITNLCPFGEVFNATRFASVYAWNRKRISNCVADYSVLYNSASFSTFKC 379
Alpha      330 .................................................. 379
Beta       330 .................................................. 379
Gamma      330 .................................................. 379
Tyrol      330 .................................................. 379
Kappa      330 .................................................. 379
Delta      330 .................................................. 379
Omicron    330 .........D...............................L.P.F.... 379

Wuhan-Hu-1 380 YGVSPTKLNDLCFTNVYADSFVIRGDEVRQIAPGQTGKIADYNYKLPDDF 429
Alpha      380 .................................................. 429
Beta       380 .....................................N............ 429
Gamma      380 .....................................T............ 429
Tyrol      380 .................................................. 429
Kappa      380 .................................................. 429
Delta      380 .................................................. 429
Omicron    380 .....................................N............ 429

Wuhan-Hu-1 430 TGCVIAWNSNNLDSKVGGNYNYLYRLFRKSNLKPFERDISTEIYQAGSTP 479
Alpha      430 .................................................. 479
Beta       430 .................................................. 479
Gamma      430 .................................................. 479
Tyrol      430 .................................................. 479
Kappa      430 ......................R........................... 479
Delta      430 ......................R.........................K. 479
Omicron    430 ..........K.....S..............................NK. 479

Wuhan-Hu-1 480 CNGVEGFNCYFPLQSYGFQPTNGVGYQPYRVVVLSFELLHAPATVCGPKK 529
Alpha      480 .....................Y............................ 529
Beta       480 ....K................Y............................ 529
Gamma      480 ....K................Y............................ 529
Tyrol      480 ....K................Y............................ 529
Kappa      480 ....Q............................................. 529
Delta      480 .................................................. 529
Omicron    480 ....A........R..S.R..Y...H........................ 529

Wuhan-Hu-1 530 STNLVKNKCVNFNFNGLTGTGVLTESNKKFLPFQQFGRDIADTTDAVRDP 579
Alpha      530 .................................................. 579
Beta       530 .................................................. 579
Gamma      530 .................................................. 579
Tyrol      530 .................................................. 579
Kappa      530 .................................................. 579
Delta      530 .................................................. 579
Omicron    530 .................K................................ 579

Wuhan-Hu-1 580 QTLE 583
Alpha      580 .... 583
Beta       580 .... 583
Gamma      580 .... 583
Tyrol      580 .... 583
Kappa      580 .... 583
Delta      580 .... 583
Omicron    580 .... 583
```

FIGURE S2. Alignment of the amino acid sequences of RBD protein of SARS-CoV-2 variants.

Shown is the sequence alignment of SARS-CoV-2 RBD (Wuhan-Hu-1, Genbank accession no. QHD43416.1) with the amino acid sequences of RBD from the indicated SARS-CoV-2 variants used in this study (GISAID initiative, https://covariants.org/) by single-letter code. Identical amino acids are denoted by dots, differing amino acids are indicated individually, and amino acid positions within the spike protein sequence are stated on the left and right sides of sequences. The A570D mutation in the SARS-CoV-2 Alpha variant was not incorporated in the Alpha construct.

FIGURE S3

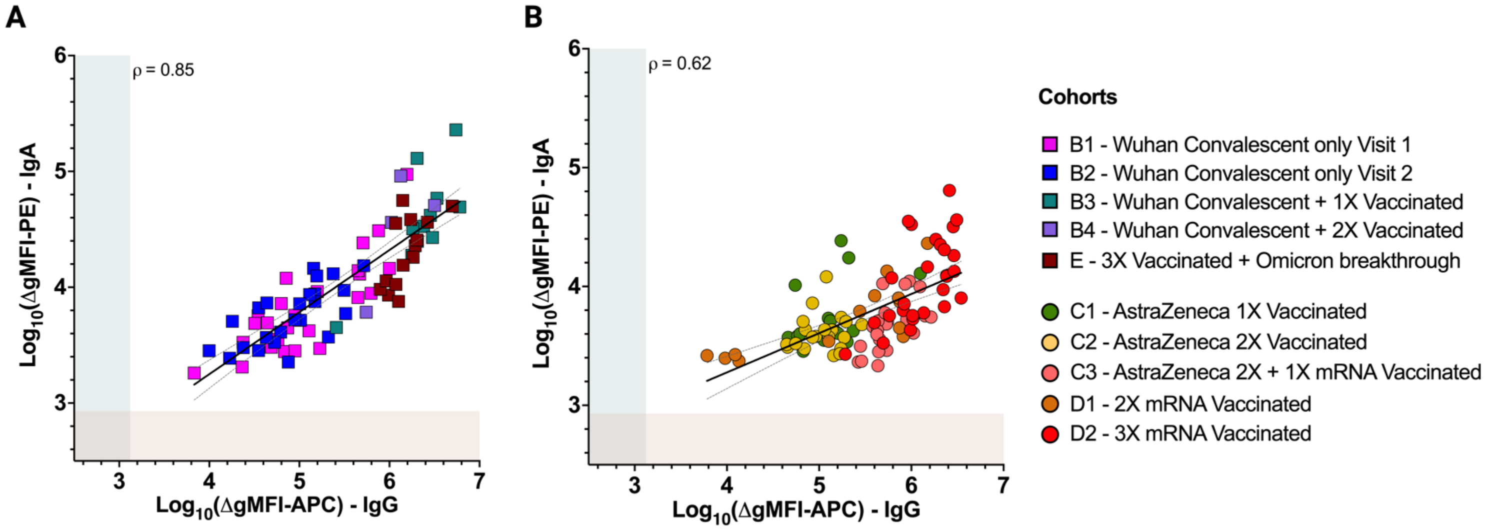

FIGURE S3. Correlation between IgG and IgA reactivity directed against RBD-Wuhan-Hu-1 as determined with the FCCA using sera from convalescent ± vaccinated or only vaccinated subjects.

Shown are the IgG and IgA correlations for convalescent ± vaccinated patients **(A)** and exclusively vaccinated individuals **(B).** X-axes show the $\log_{10}$ levels for IgG, y-axes show the respective $\log_{10}$ IgA levels. Horizontal- and vertical-colored underlays indicate FCCA thresholds. Pearson's correlation, ρ, and the linear regression line (thick black) with 95% confidence interval (dotted line) are indicated on each graph.

FIGURE S4

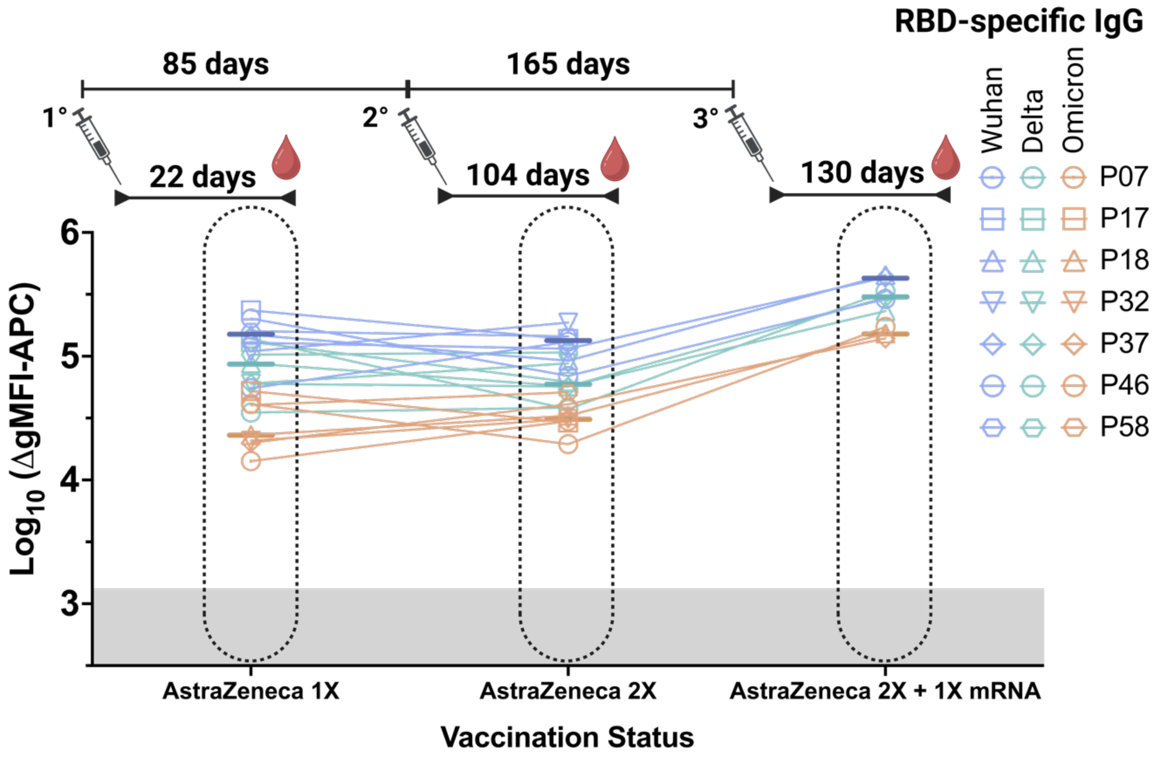

FIGURE S4. Time course of the waning of anti-RBD IgG reactivity after vaccinations. Shown are the $\log_{10}$(ΔgMFI-APC) SARS-CoV-2 RBD-specific IgG binding after vaccination of SARS-CoV-2 non-infected individuals (22-37 years of age) against the RBD-Wuhan-Hu-1 (blue), Delta (green) and Omicron (orange) HEK-293T cell lines as determined after the indicated time points. Colored horizontal lines indicate the median reactivity against RBD variants. Median days between- and post-vaccination are displayed. FCCA threshold is shaded in grey.

FIGURE S5

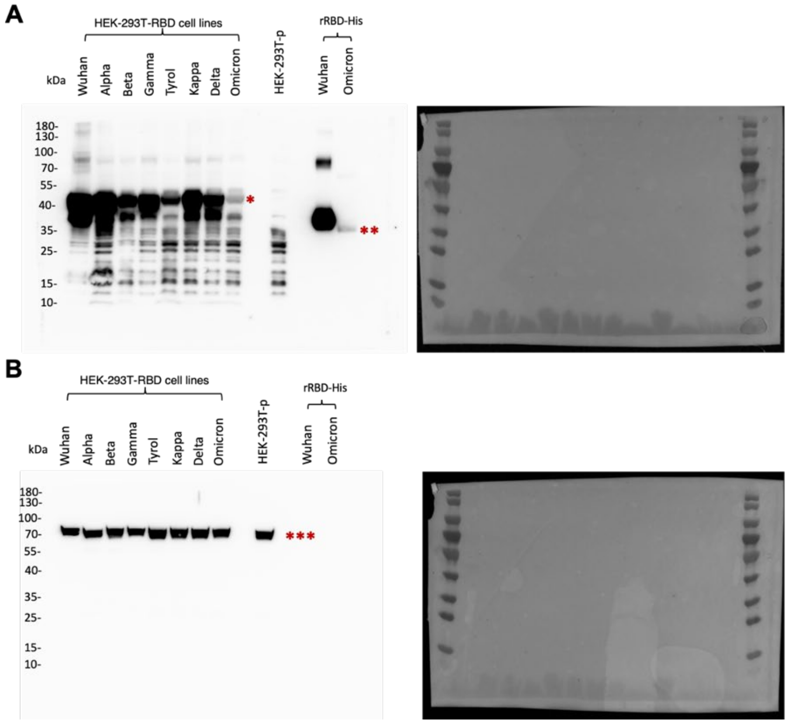

C

Densitometry		
	Blot 1	Blot 1-post-strip
	anti-RBD sera (A)	anti-calnexin (B)
RBD-Wuhan	65456	43933
RBD-Alpha	64998	42654
RBD-Beta	49312	43817
RBD-Gamma	62001	40947
RBD-Tyrol	33119	43241
RBD-Kappa	61437	43296
RBD-Delta	55764	42584
RBD-Omicron	16481	42336
HEK-293T-parental	1743	43524
rRBD-Wuhan	65142	1636
rRBD-Omicron	8388	1636
Background	1743	1636

FIGURE S5. Uncropped Western Blot images with densitometry readings. Shown in **A** and **B**, left panels, are the uncropped Western blot images of Figure 1D, probed with an anti-RBD containing highly reactive serum pool and, alternatively, anti-calnexin antibody, respectively. Moreover, the positions of the non-luminescent molecular weight markers are shown as detected with daylight (right panels). In **C**, densitometric readings measured by the ImageJ program version 1.54g of the respective bands indicated in A and B are shown. rRBD refers to recombinant RBD.

FIGURE S6

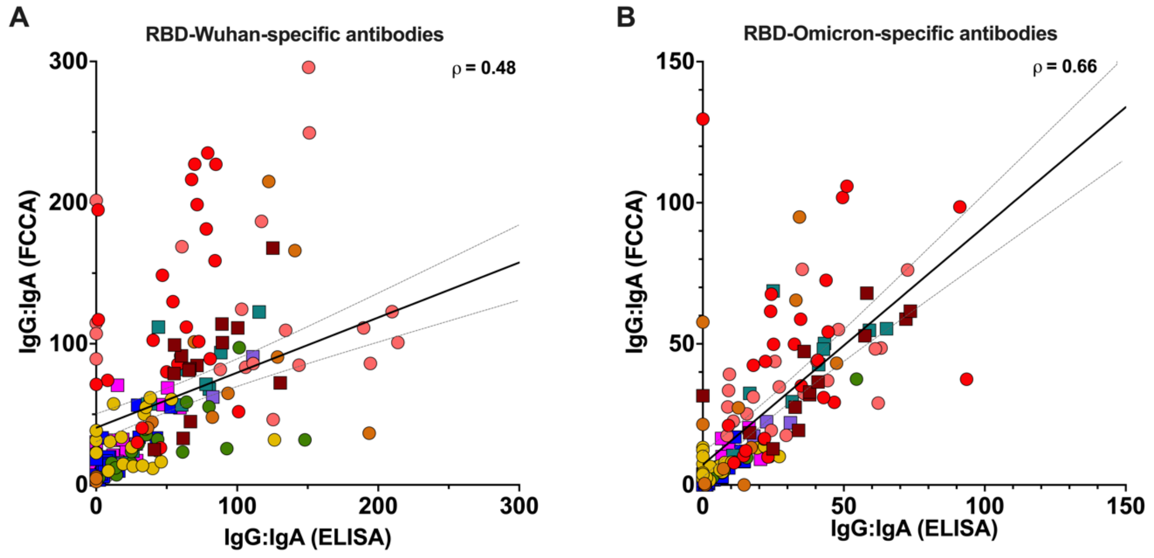

FIGURE S6. Correlation of IgG to IgA ratios between FCCA and ELISA. Shown in A and B are the RBD-specific antibody ratios between IgG and IgA against Wuhan and Omicron variants, respectively. The slope of the linear line in, A = 0.39, while in B = 0.85. IgG or IgA measurements that are below the threshold in each assay are manually assigned a ratio of 0. Pearson's correlation, ρ, and the linear regression line with 95% confidence intervals (dotted) are indicated in each graph.

FIGURE S7

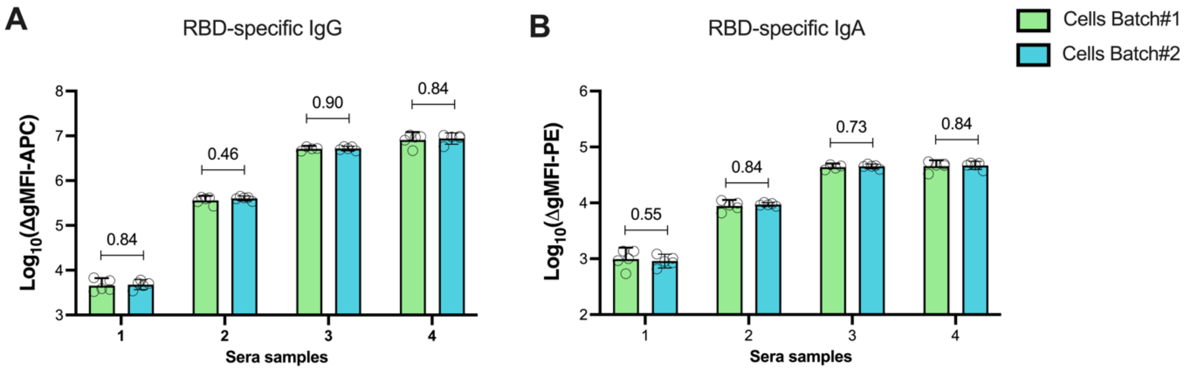

FIGURE S7. Determination of IgG and IgA anti-RBD antibody reactivity using stably expressed RBD on HEK-293T cells in the FCCA is highly reproducible.

Shown are the IgG (**A**) and IgA (**B**) reactivities of four distinct sera measured against two different batches of the same single-cell clone of RBD-Wuhan-expressing stable HEK-293T cell line, each performed in quintuplicates. Bar graphs show mean reactivities plus 95% confidence intervals. Indicated are the *p values* determined with the Mann-Whitney U test.

FIGURE S8

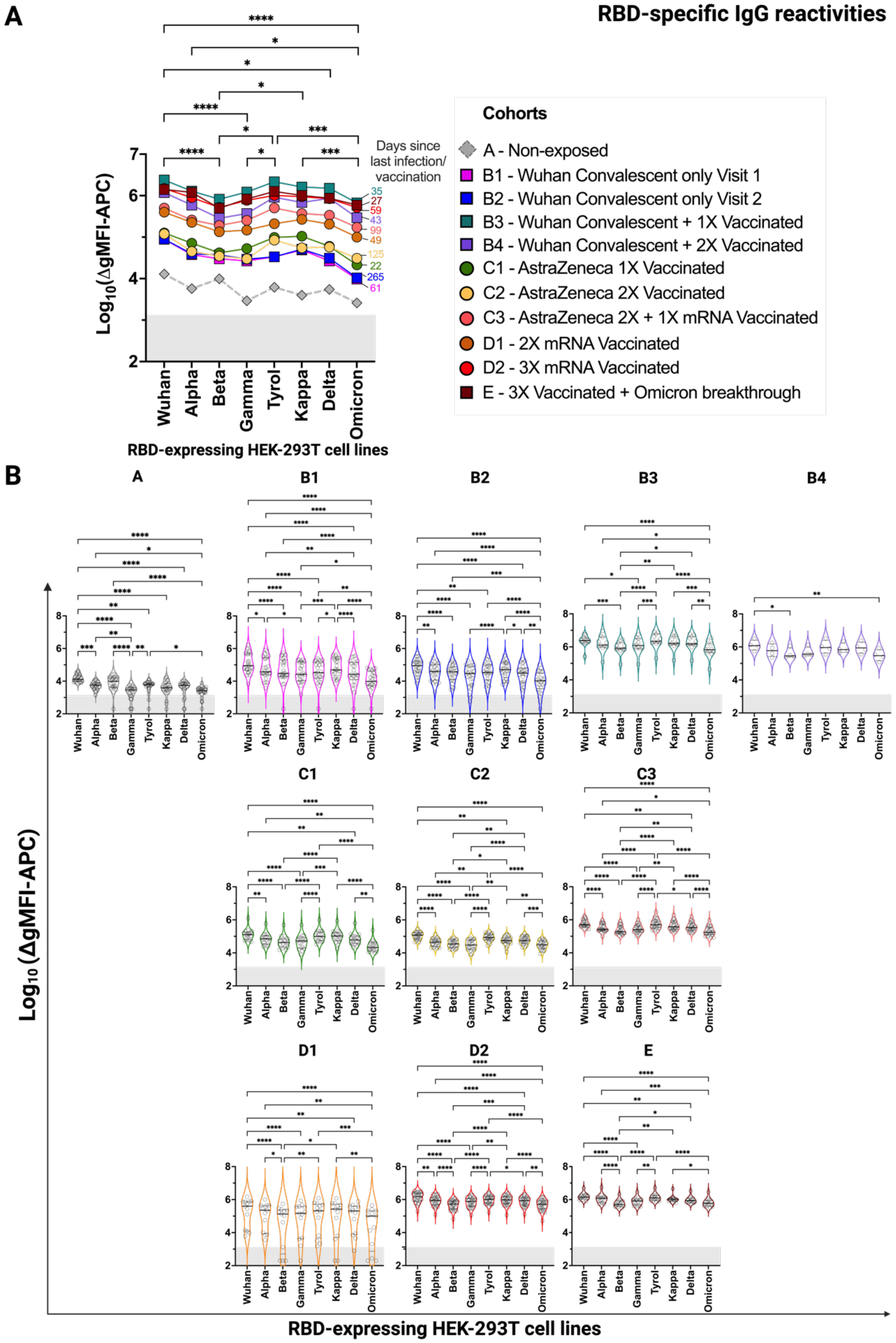
A
RBD-specific IgG reactivities
Log10(ΔgMFI-APC)
Days since last infection/ vaccination
RBD-expressing HEK-293T cell lines
Wuhan
Alpha
Beta
Gamma
Tyrol
Kappa
Delta
Omicron
Cohorts
A - Non-exposed
B1 - Wuhan Convalescent only Visit 1
B2 - Wuhan Convalescent only Visit 2
B3 - Wuhan Convalescent + 1X Vaccinated
B4 - Wuhan Convalescent + 2X Vaccinated
C1 - AstraZeneca 1X Vaccinated
C2 - AstraZeneca 2X Vaccinated
C3 - AstraZeneca 2X + 1X mRNA Vaccinated
D1 - 2X mRNA Vaccinated
D2 - 3X mRNA Vaccinated
E - 3X Vaccinated + Omicron breakthrough
B
A
B1
B2
B3
B4
C1
C2
C3
D1
D2
E
Log10 (ΔgMFI-APC)
RBD-expressing HEK-293T cell lines

FIGURE S8. SARS-CoV-2 RBD-specific IgG reactivity of groups of individuals with the indicated infection/vaccination record determined by the FCCA (multiple comparisons in all cell lines).

A shows the variant-wise comparison of median SARS-CoV-2 anti-RBD IgG antibodies (1:100) across eleven study groups, including results from a non-exposed group. Squares represent convalescent and/or vaccinated groups while circles show exclusively vaccinated groups. The median time in days since the last event (infection/vaccination) is displayed on the right side of each graph in the color of the indicated group. B represents the above information in group-wise violin plots in the color of the indicated groups. Horizontal black lines indicate the median while dotted lines show the upper and lower quartiles. IgG reactivities are displayed as log10(ΔgMFI-APC). The area indicating the FCCA threshold is shaded in grey denoting negative values. *, $p<0.05$; **, $p<0.01$; ***, $p<0.001$; ****, $p<0.0001$ as determined by Friedman test with Dunn's multiple comparison correction. In A, the statistical analysis is performed without the non-exposed group.

FIGURE S9

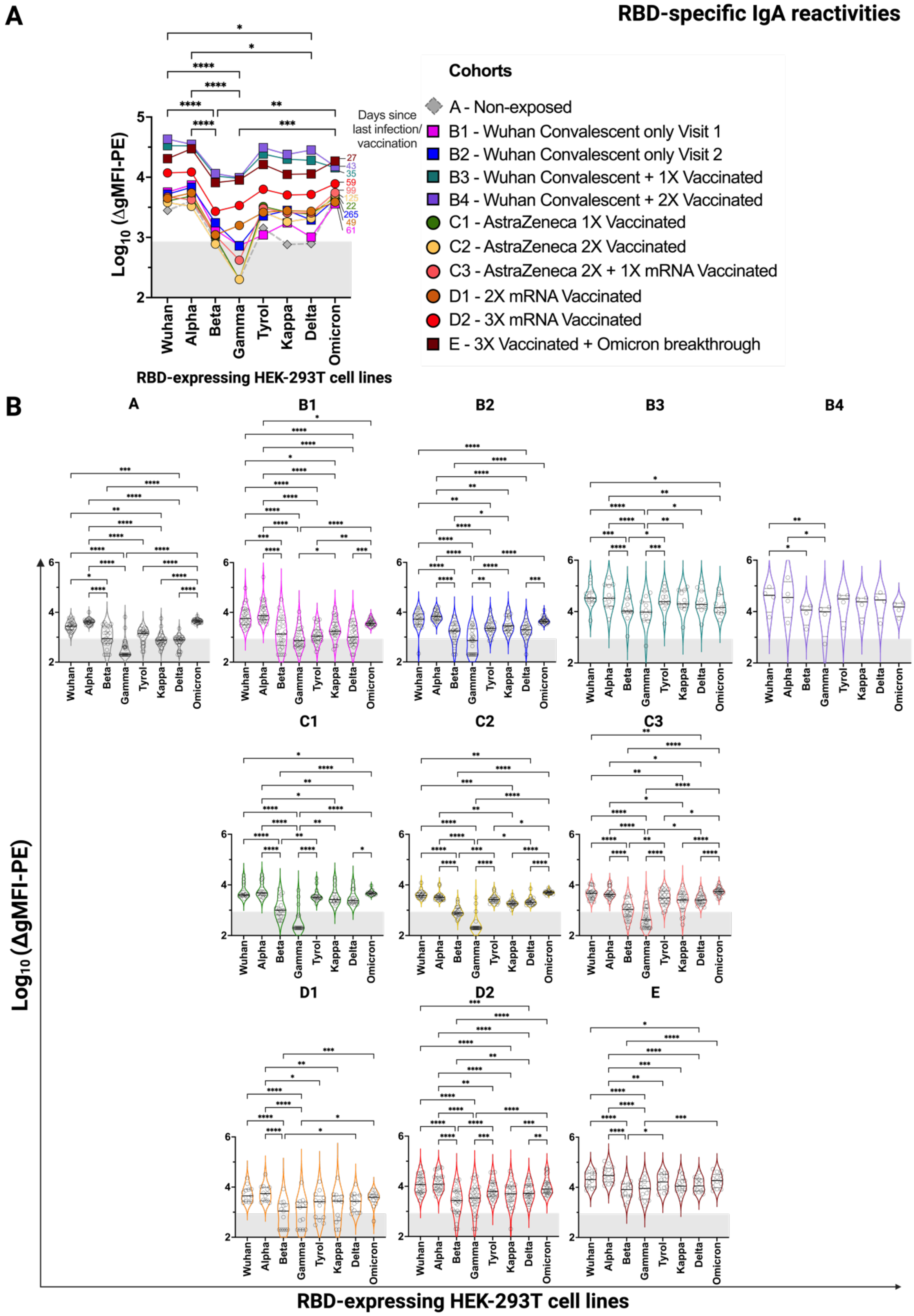
A
RBD-specific IgA reactivities
Log10 (ΔgMFI-PE)
Wuhan
Alpha
Beta
Gamma
Tyrol
Kappa
Delta
Omicron
Days since last infection/ vaccination
27
43
35
59
99
125
22
265
49
61
RBD-expressing HEK-293T cell lines
Cohorts
A - Non-exposed
B1 - Wuhan Convalescent only Visit 1
B2 - Wuhan Convalescent only Visit 2
B3 - Wuhan Convalescent + 1X Vaccinated
B4 - Wuhan Convalescent + 2X Vaccinated
C1 - AstraZeneca 1X Vaccinated
C2 - AstraZeneca 2X Vaccinated
C3 - AstraZeneca 2X + 1X mRNA Vaccinated
D1 - 2X mRNA Vaccinated
D2 - 3X mRNA Vaccinated
E - 3X Vaccinated + Omicron breakthrough
B
A
B1
B2
B3
B4
C1
C2
C3
D1
D2
E
Log10 (ΔgMFI-PE)
RBD-expressing HEK-293T cell lines

FIGURE S9. SARS-CoV-2 RBD-specific IgA reactivity of groups of individuals with the indicated infection/vaccination record determined by the FCCA (multiple comparisons in all cell lines).

A shows the variant-wise comparison of median SARS-CoV-2 anti-RBD IgA antibodies (1:100) across eleven study cohorts, including a non-exposed group. Squares represent convalescent and/or vaccinated groups while circles show exclusively vaccinated groups. The median time, in days, since the last infection ± vaccination is indicated on the right of each graph in the color of the indicated group. B represents the above information in cohort-wise violin plots in the color of the indicated groups. Horizontal black lines indicate the median while dotted lines show the upper and lower quartiles. IgA reactivities are displayed as $\log_{10}$(ΔgMFI-PE). Area indicating FCCA threshold is shaded in grey denoting negative values. *, $p<0.05$; **, $p<0.01$; ***, $p<0.001$; ****, $p<0.0001$ as determined by Friedman test with Dunn's multiple comparison correction. In A, the statistic is performed without the non-exposed group.

TABLE S1. List of primer-pairs used for the introduction of respective point mutations into the SARS-CoV-2 RBD expression constructs.

K417N	
Forward	5′-AGACAGGGAAtATTGCTGACTATAACTACAAGCTC-3′
Reverse	5′-GCCCTGGGGCAATCTGGC-3′
K417T	
Forward	5′-CAGACAGGGAcGATTGCTGACTATAACTACAAG-3′
Reverse	5′-CCCTGGGGCAATCTGGCG-3′
L452R	
Forward	5′-TACAATTATCgCTATCGGCTGTTCAGG -3′
Reverse	5′-GTTCCCGCCAACCTTGGA-3′
T478K	
Forward	5′- GCTGGATCCAagCCTTGCAACG -3′
Reverse	5′- CTGGTAGATCTCGGTAGAAATATC -3′
E484Q	
Forward	5′- CAACGGCGTCcAAGGCTTTAAC -3′
Reverse	5′- CAAGGAGTGGATCCAGCC -3′
E484K	
Forward	5′- CAACGGCGTCaAAGGCTTTAAC -3′
Reverse	5′- CAAGGAGTGGATCCAGCC -3′
N501Y	
Forward	5′- TCAGCCTACCtATGGCGTTGG -3′
Reverse	5′- AAGCCATAGCTTTGCAGG -3′

Shown in small letters are the respective nucleotides leading to point substitutions in the amino acid sequence of RBD of SARS-CoV-2 spike protein as indicated by the single-letter code (bold) and location (*italics*), according to the Human Genome Variation plasmid DNA sequence.

TABLE S1. List of SARS-CoV-2 RBD expression constructs.

Construct #	SARS-CoV-2 variant (strain designation)[1]	WHO label[1]	Pango lineage[2]	Number of mutations in RBD	Source	Cloned in plasmid vectors
1	Wuhan-Hu-1-Hu-1	-	B	-	Synthetic gene (ATG:biosynthetics)	pEAK12; pHR
2	VOC	Alpha	B.1.1.7	1	Generated by point-mutagenesis	pEAK12; pHR
3	VOC	Beta	B.1.351	3	Generated by point-mutagenesis	pEAK12; pHR
4	VOC	Gamma	P.1	3	Generated by point-mutagenesis	pEAK12; pHR
5	-	- (Tyrol) -	B.1.1.7-E484K[3]	2	Generated by point-mutagenesis	pEAK12; pHR
6	Former VOI	Kappa	B.1.617.1	2	Generated by point-mutagenesis	pEAK12; pHR
7	VOC	Delta	B.1.617.2	2	Generated by point-mutagenesis	pEAK12; pHR
8	VOC	Omicron	B.1.1.529/BA.1	16	Synthetic gene (ATG:biosynthetics)	pEAK12; pHR

VOC, variant of concern; VOI, variant of interest; WHO, World Health Organization

[1]As accessed on 31.11.23 https://www.who.int/publications/m/item/historical-working-definitions-and-primary-actions-for-sars-cov-2-variants

[2]Designations follow the PANGO (phylogenetic assignment of named global outbreak lineages) nomenclature (https://cov-lineages.org/)

[3]As accessed on 22.02.22 https://www.ages.at/en/research/wissen-aktuell/detail/distribution-and-occurrence-of-coronavirus-variants-situation-in-tyrol

TABLE S2. List of HEK-293T-based single-cell clones stably expressing SARS-CoV-2 RBD variants.

Stable transfectant #	SARS-CoV-2 variant expressing cell line	WHO label	Log_{10} (Mean)		
			Transgene expression (gMFI of FLAG-tag)	Background gMFI of isotype control Ab	Stimulation Index FLAG-tag (SI)[1]
1	RBD-Wuhan-Hu-1 SCC#1	SARS-CoV-2 (Wuhan-Hu-1)	6.8	3.3	2.0
2	RBD-N/Y SCC#6	Alpha	6.7	3.3	2.0
3	RBD-N/Y, E/K, K/N SCC#6	Beta	6.8	3.4	2.0
4	RBD-N/Y, E/K, K/T SCC#5	Gamma	7.0	3.4	2.1
5	RBD-N/Y, E/K SCC#5	- (Tyrol) -	6.7	3.4	2.0
6	RBD-L/R, E/Q SCC#6	Kappa	6.7	3.3	2.0
7	RBD-L/R, T/K SCC#8	Delta	6.5	3.3	2.0
8	RBD-Omicron SCC#8	Omicron (BA.1)	6.3	3.3	1.9

[1]SI= (log_{10} of gMFI after staining with FLAG/log_{10} of gMFI after staining with isotype control Ab). n =3 independent experiments, each performed in duplicates. SCC, Single-cell clone; MFI, mean fluorescence intensity; WHO, World Health Organization

TABLE S3. List of antibodies, staining reagents, and recombinant proteins used in this study.

**Specificity**	**Clone**	**Species**	**Conjugate**	**Source**	**Dilution**
FLAG tag	L5	Rat IgG2a, λ	PE	BioLegend	1:200
Isotype control Ab	G013C12	Rat IgG2a, λ	PE	Biolegend	1:200
F(ab')$_2$ fragment anti-human IgG, Fcγ fragment specific	polyclonal	Goat	APC	Jackson Immuno	1:100
AffiniPure anti-human serum IgA, α chain specific	polyclonal	Goat	PE	Jackson Immuno	1:100
Human heparin plasma/sera	-	-	-	-	1:100 (FCC Assay)<br>1:200 (IgG ELISA)<br>1:100 (IgA ELISA))
Human IgG	G18-145	Mouse IgG1, κ	HRP	BD Bioscience	1:2000 (ELISA)<br>1:8000 (Western Blot)
Human IgA1/IgA2	G18-1	Mouse IgG1	Purified	BD Bioscience	1:500
Mouse IgG1	polyclonal	Sheep	HRP	Cytiva	1:1000
Human Calnexin	C5C9	Rabbit IgG	Purified	Cell Signaling	1:2000
Rabbit Ig	polyclonal	Goat	HRP	Agilent	1:8000

Zombie Aqua™	-	-	BV520	BioLegend	1:200
ACE2, hFc	-	Human	Purified	GenScript	1:650
Spike RBD-Wuhan-Hu-1-Avi-His	-	-	Purified	GenScript	2 μg/mL (ELISA)<br>50 ng (Western Blot)
Spike RBD-His Delta (L452R, T478K)	-	-	Purified	SinoBiological	2 μg/mL (ELISA)
Spike RBD-His B.1.1.529 (Omicron)	-	-	Purified	SinoBiological	2 μg/mL (ELISA)<br>50 ng (Western Blot)
